# Immortalized B Cells Transfected with mRNA of Antigen Fused to MITD (IBMAM): An Effective Tool for Antigen-Specific T-Cell Expansion and TCR Validation

**DOI:** 10.3390/biomedicines11030796

**Published:** 2023-03-06

**Authors:** Zhe Wang, Tiantian Zhang, Aaron Anderson, Vincent Lee, Szymon Szymura, Zhenyuan Dong, Benjamin Kuang, Elizabeth Oh, Jingwei Liu, Sattva S. Neelapu, Larry Kwak, Soung-chul Cha

**Affiliations:** 1 Toni Stephenson Lymphoma Center, Hematologic Malignancies Research Institute, Department of Hematology and Hematopoietic Cell Transplantation, City of Hope, Duarte, CA 91010, USA; 2 Department of Lymphoma and Myeloma, Division of Cancer Medicine, The University of Texas, MD Anderson Cancer Center, Houston, TX 77030, USA

**Keywords:** immunomonitoring, antigen-specific T cells, MITD, B cell immortalization, mRNA, novel platform, CMV, TCR validation and discovery

## Abstract

Peripheral mononuclear blood cells (PBMCs) are the most widely used study materials for immunomonitoring and antigen-specific T-cell identification. However, limited patient PBMCs and low-frequency antigen-specific T cells remain as significant technical challenges. To address these limitations, we established a novel platform comprised of optimized HLA-matched immortalized B cells transfected with mRNA of a prototype viral or tumor antigen conjugated to MHC class-I trafficking domain protein (MITD) to increase the efficiency of epitope expression in antigen-presenting cells (APCs) essential to expanding antigen-specific T cells. When applied to CMV as a model, the IBMAM platform could successfully expand CMV-specific T cells from low-frequency CMV PBMCs from seropositive donors. Additionally, this platform can be applied to the validation of antigen specific TCRs. Together, compared to using APCs with synthesized peptides, this platform is an unlimited, highly efficient, and cost-effective resource in detecting and expanding antigen-specific T cells and validating antigen-specific TCRs.

## 1. Introduction

Immunomonitoring of antigen-specific T cells is critical for evaluating cellular immune function and essential in understanding immune-mediated diseases, and antigen-specific immunotherapy development [1]. Due to their accessibility in ex vivo studies, peripheral mononuclear blood cells (PBMCs) have been the most widely used study materials for detecting antigen-specific T cells and their responses. However, the low-frequency of antigen-specific T cells in PBMCs has made the study technically difficult and is an obstacle that remains to be solved. Because graded epitope-specific T-cell responses are generated over a wide range of epitope levels, epitope expression levels have been shown to dramatically influence the magnitude of the responding antigen-specific T-cell population [2]. Therefore, increasing epitope expression level is an appealing method for expanding low-frequency antigen-specific T cells. The same rationale of increasing major histocompatibility complex MHC-I and MHC-II antigen presentation has been applied in the development of viral and neoantigen vaccines [3]. Therefore, to increase epitope expression levels, we established a novel platform using immortalized B cells, which serve as antigen-presenting cells (APCs) transfected with the mRNA of antigen conjugated with the MHC class I trafficking domain (MITD). We hypothesize that optimizing APCs with high level epitope expression leads to efficient antigen-specific T-cell expansion from PBMCs. While APCs present epitopes for TCR recognition and T-cell activation, we further hypothesize that this novel platform is useful in TCR confirmation and discovery. 

In this novel platform, the mRNA of the antigen, MITD, and APCs are the three key components essential for increased epitope expression. The application of mRNA transfection for immunological studies and gene therapies by simple electroporation protocol has been proven to be an extremely efficient tool. For instance, in neoantigen studies, the mRNA encoding candidate neoepitopes were electroporated into dendritic cells (DCs) for priming of autologous antigen-specific T cells. In comparison to using synthesized peptides to be pulsed to DCs, the usage of mRNA of epitope minigenes appears to be more convenient and enables high throughput screening of many candidate neoepitopes with increased efficiency [4]. Furthermore, the full-length mRNA of the tumor antigen is a useful method for presenting antigens to T cells when the specific epitopes are unknown and cannot be synthesized as peptides. Therefore, in this study, we investigate the use of full-length mRNA transfection for naturally processed epitope presentation.

Highly conserved amino acid sequences or motifs were found in the cytoplasmic domains of some integral membrane proteins, which are essential in controlling their trafficking and intracellular localization. These motifs, which are often leucine- or tyrosine-based, act as the intracellular “addressins” to be recognized by other trafficking components and direct the localization of these proteins to the desired compartments [5]. An array of trafficking motifs, such as invariant chain, lysosome-associated membrane proteins (LAMPs), and MITD, have been used to attach to antigens, aiming to enhance antigen trafficking through MHC-I or MHC-II intracellular compartments [6,7]. It is noteworthy that among these motifs, MITD coupling to antigens has been shown to reroute the antigen presentation and translocate into diverse antigen processing compartments harboring both MHC-I and MHC-II molecules [5,6]. The mutation of the cytoplasmic tyrosine of MITD has been demonstrated to drastically reduce the localization of MHC-I in endosomal and lysosomal compartments of APCs.

Monocyte-derived DCs (moDCs) have been widely used as potent APCs, due to their high phagocytic activity to capture antigens for immune presentation and the capability of cross-presenting exogenous antigens on class I MHC complexes [8,9]. However, the limitations in their practical usage are the requirement of isolating CD14+ monocytes from PBMCs every time prior to use and the need to induce their differentiation and maturation into moDCs with granulocyte macrophage-colony-stimulating factor (GM-CSF) plus interleukin (IL)-4. B-cell immortalization has provided an attractive alternative method for generating stable and easily expanding B cells as APCs which can be an unlimited resource. With transfection of retrovirus expressing BCL-6 and BCL-XL genes, the peripheral blood memory B cells can be converted to germinal center-like B cells and maintained for long culture [10,11]. B cells immortalized in such a manner and loaded with neopeptides or proteins have been shown to possess the capability to stimulate antigen-specific CD4+ T cells [12,13]. 

In this study, we have established a novel platform where autologous-immortalized B cells processing the mRNA of antigens linked to MITD (IBMAM) are used for antigen-specific T-cell expansion and TCR validation. We demonstrated that in immortalized B cells, MHC class II or MHC class I molecules were naturally processed and effectively presented epitopes to CD4+ T cells or CD8+ T cells when antigens were fused with MITD molecules. The optimal methodology for B-cell immortalization has been further explored. While we have proven the efficacy of MITD in improving antigen presentation, here we further tested an array of MITD variants and compared their efficacies. In addition, we tested the optimal arrangement of epitope minigenes in mRNA processing and antigen presentation. When applying this platform to antigen-specific T-cell expansion, we developed two pathways based on two conditions of whether the antigen-specific T cells can be initially detected from the PBMC samples. Using CMV seropositive normal healthy donors’ PBMC samples, we show that our platform can successfully expand CMV-reactive T cells, regardless of whether the CMV-specific T cells are initially detectable. Moreover, we further demonstrate the capability of our platform in validating TCR reactivity and selecting reactive TCRs, which can be applied in TCR discovery and screening. 

## 2. Materials and Methods

### 2.1. Normal Healthy Blood, Patients, and Biospecimens

Normal healthy donor peripheral blood mononuclear cells (PBMC) were provided by the Michael Amini Transfusion Medicine Center at City of Hope (IRB: 15283). PBMC were also obtained from lymphoma patients who were enrolled in the institutional review board (IRB)-approved protocol at MD Anderson Cancer Center (Protocol 2009-0465, NCT01209871).

### 2.2. Cell Culture [B Cells, Feeder Cells, T Cells, Jurkat TCRαβ-KO (CD4+, CD8+)]

B cells of four CMV-seropositive healthy donors (#946, #647, #739 and #673 from City of Hope blood donor center), lymphoma patients (Pt. 1, Pt. 2, Pt. 3, Pt. 4, Pt. 5, Pt. 6, and Pt. 7 from MD Anderson Cancer Center) were used (Supplemental Table S1). Immortalized B cells, BJ feeder cell lines (from Dr. Neelapu at MD Anderson), T cells of four healthy donors, CMV specific CD8+ T cells (asteria comp), and TCRαβ-KO (CD4+CD8+) cells (Promega Corporation, Madison, WI, USA) were used in cell experiments. B cells, feeder cells, and T cells were cultured in RPMI-1640 medium (Gibco, Billings, MT, USA) supplemented with GlutaMAX-HEPES (Gibco), 10% Human AB serum (Valley Biomedical), and 1% L-glutamine (Gibco). Jurkat TCRαβ-KO (CD4+CD8+) cells were cultured in RPMI 1640 (with L-glutamine and HEPES 10% FBS) supplemented with 400 µg/mL hygromycin B, 1 mm sodium pyruvate, 0.1 mm MEM nonessential amino acids, and 10 μg/mL blasticidin S HCl (ThermoFisher, Waltham, MA, USA). All the cells were cultured at 37 ℃ with 5% CO_2_ and 100% humidity.

### 2.3. Plasmid DNA

In vitro transcription (IVT) constructs were designed using the pMRNAxp vector (System Biosciences) as a template, modified by introduction of a signal peptide fragment (SP), linker (G_4_S)_2_, an antigen gene, V5 tag (GKPIPNPLLGLDST), the MITD including the stop-codon, and a 120-bp poly (A) tail [14] (named pMITD-WT) [6]. Antigen genes of interest were cloned into the pMITD-WT vector containing *Bgl*II and *Xho*I restriction sites. The cDNA of CMV pp65 and HIV Nef were purchased from OriGene. To make the pCMV-MITD-WT plasmid, the full-length CMV pp65 gene was cloned into the pMITD-WT vector from cDNA by PCR using pp65-specific primers: Fwd-pp65 (5′-GGT GGC GGT GGG AGC AGA TCT GCC GTG TTT AGC CGG GGA) and Rev-pp65 (5′-AGG GAT AGG CTT ACC CTC GAG CAT ATG GCC TCT ATG) containing *Bgl*II and *Xho*I restriction sites. To make pNef-MITD-WT plasmid, the full-length HIV Nef gene was cloned into pMITD-WT vector from the cDNA of Nef by PCR using Nef-specific primers: Fwd-Nef (5′-GGT GGC GGT GGG AGC AGA TCT ATG GGT GGC AAG TGG) and Rev-Nef (5′-AGG GAT AGG CTT ACC CTC GAG TAT GCA GCA TCT GAG GGC). The pNY-ESO-1-MITD-WT plasmid, which is the pMITD-WT vector containing the full-length NY-ESO-1 gene, was purchased from Geneart. Similarly, the pLAMP2A-MITD-WT plasmid, which is the pMITD-WT vector containing the LMP2A core epitope (ESEERPPTPY: HLA-A*0101) was synthesized as gBlocks (IDT).

The pGFP and pGFP-MITD-WT were also produced using the method described above. To make the GFP-MITD-WT and pGFP plasmids, a *Bgl*II/*Xho*I -site-flanked eGFP fragment, amplified from pEGFP-C1 vector (Clontech), was cloned into the pMITD-WT vector using eGFP sense specific primers: Fwd-GFP 5′-GGT GGC GGT GGG AGC AGA TCT GTG AGC AAG GGC GAG GAG; Rev-GFP Stop antisense 5′-AGG GAT AGG CTT ACC CTC GAG TCA CTT GTA CAG CTC GTC CAT; Rev-GFP-MITD 5′-AGG GAT AGG CTT ACC CTC GAG CTT GTA CAG CTC GTC CAT). pGFP plasmids were constructed using a Rev-GFP stop antisense primer to exclude MITD. 

The short-2 (S2) gene sequence was designed using a combination of CMVpp65 core peptide coding genes (NLVPMVATV_aa 495–503_: HLA-A*0201) and the other core peptide coding gene (PLKMLNIPSINVHHYPSAAERKH_aa 337–359_.: HLA-DRB1*0701) [15]. The S2 design combined the MHC-I and II epitopes for CMV pp65 with a disulfide linker ((G_4_S)_2_) in between. The short-10 (S10) sequence was modified to repeat the S2 gene design five times. The long-2 (L2) gene sequence adds three amino acids to the left and right side of the MHC-I and II epitopes, respectively, and then combines the modified MHC-I and II epitopes as described for the S2 gene sequence (Supplementary Table S1). For mRNA expression, S2, L2, and S10 genes were synthesized as gBlocks (IDT) and cloned into the pMITD-WT vector containing *Bgl*II and *Xho*I restriction sites. The vector templates were generated, containing MITD-wild type (MITD-WT) and respectively S2 synthesis gene (S2-MITD-WT), L2 synthesis gene (L2-MITD-WT), and S10 synthesis gene (S10-MITD-WT).

The other set of four CMV-MITD constructs were generated, containing either the CMVpp65-full length gene and respectively MITD-WT (CMV-MITD-WT), the MITD-S335E (CMV-MITD-S335E), the MITD-Y320E (CMV-MITD-Y320E) or the MITD-Y320E/S335E (CMV-MITD-Y320E/S335E) genes. The cDNA encoding MITD with a CMV pp65 epitope (pCMV-MITD-WT) was used as a template to generate point mutations at S335 and/or Y320. To make a point mutation to glutamic acid of MITD from tyrosine amino acid positioned at 320 aa, the MITD-Y320E gene was synthesized 229nt by changing TAC to GAG and cloned into the *Pst*I (*CTGCAT*) and *Bam*HI (*GGATCC*) site of the pCMV-MITD-WT vector. To make a point mutation to glutamic acid of MITD from serine amino acid positioned at 335 aa, the MITD-S335E gene was synthesized 229nt by changing TCT to GAG and cloned into the *Pst*I (*CTGCAT*) and *Bam*HI (*GGATCC*) site of the pCMV-MITD-WT vector. For double mutations, the MITD-Y320E/S335E gene was synthesized 229nt by changing TAC to GAG and TCT to GAG and cloned into *Pst*I (*CTGCAT*) and *Bam*HI (*GGATCC*) site of the pCMV-MITD-WT vector (Supplemental Table S2). 

The entire sequences of two CMV pp65-specific TCRβ-2A-TCRα (TCRCD8-CMV#8, TCRCD4-CMV#4 [15], Supplemental Table S1) were synthesized by Integrated DNA Technologies and were ligated into the PCI vector via the in-fusion cloning kit.

### 2.4. Generation of Immortalized B Cells

To establish autologous immortalized B cells as antigen presenting cells in vitro, we first introduced the genes encoding BCL-6 and BCL-XL into B cells, using retroviral vectors as previously described [10]. To create recombinant retrovirus for retroviral constructs, the pRetro-X-IRES-ZsGreen1 vector (TaKaRa Bio, Kusatsu-shi, Japan) was used to ligate the cDNA encoding BCL-6 and BCL-XL proteins. The retroviral plasmids were subsequently transfected into the GP2-293 packaging cell line expressing gag and pol proteins (TaKaRa Bio). A separate plasmid, pCMV-GALV-MTR (Addgene), was employed to provide the envelope protein, as per the manufacturer’s instructions. After four days of transfection, the retroviral supernatant was harvested, concentrated using Retro-X concentrator, and stored as cell-free aliquots at −80 °C. To generate autologous immortalized B cells, primary B cells were extracted from PBMCs utilizing the EasySep Human B cell isolation kit (Stemcell Technologies Inc., Vancouver, BC, Canada). Through co-culturing with fibroblastic BJ feeder cells that express CD40L and secrete IL-21 for 48 h at 37 °C, the isolated primary autologous B cells were activated before transduction. The activated primary autologous B cells were then ex vivo transduced with the BCL-6/BCL-XL recombinant retrovirus using the human fibronectin fragment CH-296 transduction protocol as described (RetroNectin; TaKaRa Bio) [16]. Transduction efficiency was measured by flow cytometry, and GFP+, CD19+ B cells were sorted out and freshly cultured with engineered BJ feeder cells expressing CD40L and secreting IL-21.

### 2.5. In Vitro Transcription of Antigens mRNA and Electroporation of the mRNA to Immortalized B Cells

The vector template was used for the generation of RNA encoding CMVpp65, CMV-MITD-WT, Nef-MITD-WT, S2-MITD-WT, L2-MITD-WT, S10-MITD-WT, CMV-MITD-S335E, CMV-MITD-Y320E, and CMV-MITD-S335E/Y320E for in vitro transcription (plasmid DNA production process shown as above). Each construct contains the signal peptide (SP), MHC class I trafficking domain (MITD), and 120bp poly(A) tail. The pMRNA-120bp-based plasmids were linearized with restriction enzyme *Not*I and used as templates for in vitro transcription (IVT). After purification, IVT was performed with the T7 polymerase using the mMESSAGE mMachine Ultra T7 kit (ThermoFisher, Waltham, MA, USA). RNA quality and concentration were assessed by agarose/formaldehyde gel electrophoresis and spectrophotometry, respectively. RNA was transfected into 1 × 10^6^ immortalized B cells at a concentration of 30 μg/10^6^ cells using the neon electroporation system (pulse voltage at 1150 V, pulse width at 30 ms, pulse number at 2). 

### 2.6. HLA Genotyping

DNA was isolated from frozen PBMCs with the DNeasy Blood & Tissue Kit (Qiagen Benelux, Venlo, The Netherlands). DNA concentration was determined by NanoDrop (Thermo Fisher Scientific, Waltham, MA, USA) and samples were concentrated to 50 ng/μL. HLA class I and II loci (HLA-A, -B, -C, -DRB1, -DQA1, -DQB1) were genotyped at four-digit resolution in City of Hope Histocompatibility Laboratory [17].

### 2.7. Rapid Expansion Protocol (REP)

For in vitro expansion, CD4+ T cells were isolated from PBMCs using the EasySep Human CD4+ T-cell isolation kit (Stemcell Technologies). Following a rapid expansion protocol (REP) as previously described, isolated cells were expanded. [18]. In the presence of irradiated, allogeneic PBMC feeder cells at a 200:1 ratio of feeder cells to patients’ CD4+ T cells, the REP utilized OKT3 (anti-CD3) antibody (Ortho Biotech, Raritan, NJ, USA) and IL-2 (100 IU/mL). By apheresis, PBMC feeder cells were achieved from three normal volunteers, which were thawed, washed, and resuspended in 25 mL of CTL media (RPMI 1640, GlutaMAX-HEPES + 10% Human AB serum; ThermoFisher), and irradiated (50 Gy). We combined, mixed and aliquoted PBMC feeder cells (1 × 10^8^), OKT3 antibody (30 ng/mL), 25 mL of CTL media, and CD4+ T cells (0.5 × 10^6^) to a 25 cm^2^ tissue culture flask. The flasks were kept upright and incubated at 37°C in the presence of 5% CO_2,_ and IL-2 at 100 IU/mL was added again on day 2. On day 5, the culture supernatant was aspirated, leaving the cells at the bottom of the flask, and replaced with a CTL media containing 100 IU/mL IL-2. From day 6 onwards, the concentration of cells was measured, and as required to maintain cell densities of around 1 × 10^6^ cells/mL they were split into additional flasks or transferred to 75 cm^2^ tissue culture flasks with additional medium containing 100 IU/mL IL-2. The cells were harvested from the culture flask and cryopreserved for future experimental analysis after approximately 14 days of the REP.

### 2.8. ELISA

Human IFNg Uncoated ELISA Kit (Invitrogen, Waltham, MA, USA) and human IL-21 ELISA kits (Biolegend, San Diego, CA, USA) were used for the measurement of IFNg secretion by T cells and IL-21 secretion by BJ feeder cells. For the IFNg secretion assay (as described below), the supernatant was saved after experiments. For IL-21 detection, the supernatant was collected after 3–5 day of BJ feeder cell culturing. The ELISA procedures were performed according to the manufacturer’s instructions. Experiments were carried out either in duplicate or triplicate.

### 2.9. Flow Cytometric Analysis

To examine the frequency of CD19+GFP+ immortalized cell (transduction efficiency), B cells were transfected with the genes encoding BCL-6 and BCL-XL, then stained with anti-CD19 on day 14, 21, 30 of post-transfection and subsequently analyzed using a FACS LSRFortessa™ (BD Biosciences; Franklin Lakes, NJ, USA). To test the biomarkers of the immortalized B cells, immortalized B cells were stained with anti-CD19, anti-HLA-ABC, anti-HLA-DR, anti-CD40, anti-CD80, anti-CD83, and anti-CD86. Flow cytometric analysis was performed on a FACS LSRFortessa™ analytical flow cytometer using BD FACS Diva (BD Biosciences) or FlowJo software (FlowJo, LLC, Ashland, OR, USA).

### 2.10. Immunofluorescence Staining

The immortalized B-cell culturing microenvironment and mRNA expression were visualized using Zeiss LSM 880 with Airyscan. All images were processed using ZEN V3.4 software (Zeiss, Oberkochen, Germany‎). For visualization of immortalized B-cell culturing microenvironment, immortalized B cell expressing red fluorescence protein (RFP) was stained with anti-CD40L mAb. Isotypic antibody was used for negative control.

### 2.11. Jurkat T-Cell Activation Bioassay

TCRαβ-KO (CD8+, CD4+) Jurkat T-cell line (Promega), which expresses a luciferase reporter driven by a TCR pathway-dependent promoter, was used for testing TCR specificity and assess functional avidity to epitope via the antigen processing in immortalized B cells. The PCI mammalian vector (Promega) containing sequences of CMV pp65-specific TCRs with HLA class I restriction (recognizing pp65 aa 495–503) or with HLA class II restriction (recognizing pp65 aa117–139) were constructed, designated as PCI-TCR-CMV-CD8 and PCI-TCR-CMV-CD4. The PCI-TCR-CMV-CD8 and PCI-TCR-CMV-CD4 were transfected into 2 × 10^6^ TCRαβ-KO Jurkat T cells at a concentration of 30 μg/10^6^ cells on day 1 using neon electroporation system (pulse voltage at 1350 V, pulse width at 10 ms, pulse number at 3) and incubated for 48 h. On day 3, the TCR expression was detected using flow cytometric analysis and Jurkat cells were washed using PBS and resuspended in the assay buffer (RPMI + 1% FBS) at a density of 1 × 10^6^ cells/mL. The HLA-matched immortalized B cells were transfected with the desired mRNAs and incubated in the assay buffer at a final density of 2 × 10^6^ cells/mL. About 40 μL of Jurkat cells and 40 ul of transfected immortalized B cells were mixed (at a Jurkat:B cell ratio of 1:2) and incubated in the 96-well plate at 37 °C. After 24 h, the desired amount of reconstituted Bio-Glo-NL™ Reagent was prepared by combining one volume of substrate with 50 volumes of buffer, and 80 μL of the reconstituted Bio-Glo-NL™ Reagent was added to the assay plate wells of incubated Jurkat and immortalized B cells. After 10 min incubation at room temperature, the luminescence was measured using a plate reader with glow-type luminescence reading capabilities.

### 2.12. IBMAM Stimulation of PBMCs

Irradiated washed autologous immortalized B cells (1 × 10^6^) were transfected with antigen mRNA (30 μg/10^6^ cells) and were cocultured with PBMCs (10 × 10^6^) in CTL media containing 10% human AB serum in 1 well of a 24-well plate. Additional IL-2 was added at 100 IU/mL and IL-7 was added at 10 ng/mL on day 2 and day 5. For pathway 2, cultures were restimulated with irradiated washed autologous immortalized B cells (1 × 10^6^) and then transfected with antigen mRNA (30 ug/10^6^ cells) and IL-2 on day 8. Additional IL-2 was added at 100 IU/mL and IL-7 was added at 10 ng/mL on day 9 and day 12. After two separate stimulations 7 days apart (day1 and day8), the CD4+ T cells were isolated on day 15. 

### 2.13. IBMAM Detection of Antigen-Specific T Cells (Cytokines Secretion Assay)

The T-cell specific reactivity to antigens was tested using the recognition assays, which were carried out in 96-well plates where 1 × 10^5^ target cells (autologous immortalized B cells transfected with antigen mRNA (30 μg/10^6^ cells)) and 1 × 10^5^ of the isolated, stimulated T cells were cocultured for 48 h in 200 µL at 37 °C in 5% CO_2_. The superantigen SEB (Staphylococcus aureus, Enterotoxin Type B) served as a positive control, and a “No antigen” control group was also included. Supernatants were harvested, and cytokines were assayed by ELISA or cytometric bead assay.

### 2.14. Expansion of Antigen-Specific T Cells 

About 1 × 10^5^ target cells (autologous immortalized B cells transfected with antigen mRNA (3 μg/1 × 10^5^ cells)) and 1 × 10^5^ of the isolated, stimulated T cells were co-cultured in 96-well plates for 24 h in 200 µL at 37 °C in 5% CO_2_. The superantigen SEB served as a positive control, and a “No antigen” control group was also included. Subsequently, cells were stained using the IFNg capture kit (Miltenyi Biotec, Bergisch Gladbach, Germany) according to manufacturer’s guidelines plus anti-CD4 antibody (BD Pharmingen). Single, live IFNg producing CD4+ T cells were sorted by a BD FACSAria™ Fusion cell sorter (BD Biosciences) and collected in 5 mL tubes. Sorted CD4+T cells were expanded using a REP as previously described. After 14 days of initiation of the REP, cells were harvested from the culture flask and were used for cytokines secretion assay.

### 2.15. Statistical Analysis

Statistical analysis was performed using Graphpad Prism software (V9.4.1). Data are shown as the means ± standard deviations. Statistical analysis for individual experiments is indicated as described in figure legends. Statistical significance was determined using a two-sided Student’s *t*-test when only two groups were analyzed unless specifically noted. * *p* < 0.05.

## 3. Results

### 3.1. Characterization of Immortalized B Cells

**Figure 1:**
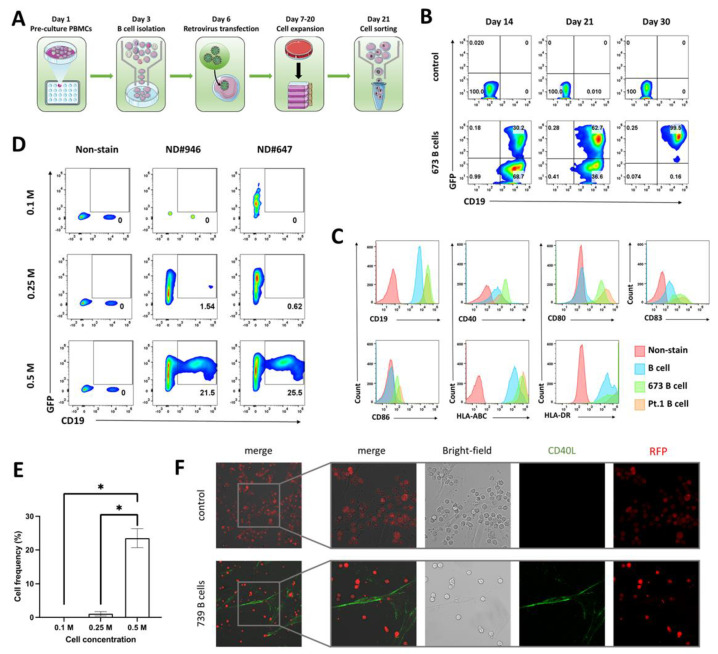
Characterization of B-cell immortalization. (**A**) General schema for B-cell isolation and immortalization. The PBMCs from NDs were grown at 10^7^/mL for 2 days before the B cells were isolated and co-cultured with BJ feeder cells. At day 6, the expanded B cells were transfected with retrovirus expressing BCL-6/BCL-XL and GFP-reporter genes, and cultured with supplementation of CD40L, IL-2, and IL-4. After overnight incubation, the transfected B cells were transferred to new plates containing feeder cells. Following 7–20 days proliferation, the GFP+CD19+ cells were sorted from the transfected B cells. (**B**) FACS analysis of ND#673 B-cell immortalization. After retroviral transfection, percentage of CD19+GFP+ cells were identified at day 14 and 21, with CD19+GFP+ cells subsequently isolated from expanded B-cell culture. The isolated cells were proliferated with BJ feeder cells and confirmed at day 30 for CD19 and GFP expression. (**C**) Immortalized B-cell biomarker identification. Following co-culturing with feeder cells, the immortalized B cells from ND#673 and lymphoma patient 1 (Pt. 1) were subjected to flow cytometric analysis for expression of B-cells biomarkers, including CD19, CD40, CD80, CD63, and CD86. B cells from ND PBMCs with no feeder cell stimulation served as the control cells. (**D**,**E**) Cell quantity optimization for B-cell immortalization. Different quantities (respectively 0.1, 0.25 and 0.5 million) of B cells isolated from PBMCs of ND1 and ND2 were immortalized with above mentioned protocol, with GFP and CD19 expression analyzed at day 10. * (*p* < 0.05). (**F**) Confocal imaging showing immortalized B-cell culturing microenvironment. Immortalized B cells engineered to express red fluorescence protein (RFP) were cultured with feeder cells for extensive proliferation. The feeder cells were stained with anti-CD40L. See also Figure S1.

Dendritic cells (DCs) have traditionally been used as antigen-presenting cells. However, using DCs can be burdensome as it requires the isolation of immature DCs from peripheral blood mononuclear cells (PBMCs) each time, induction of differentiation into mature DCs, and supplementation of CD14 and GM-CSF for cell growth. In contrast, immortalized B cells provide an unlimited, “ready-to-use” resource that does not require induction for maturation. Previous research has shown that the expression of oncogene BCL-6 is able to inhibit B-cell differentiation into plasma cells and that BCL-6 transduced cell lines can express a phenotype consistent with germinal center B cells [10]. Another anti-apoptotic oncogene, BCL-XL, when co-expressed in BCL-6 transduced B cells, can exert a remarkable effect in prolonging the cell proliferation and preventing cell death, particularly in response to CD40 stimulation [19]. To establish our immortalized B cells, B cells were isolated from normal healthy donor (ND) PBMCs and transfected with retrovirus expressing BCL-6 and BCL-XL genes, as well as GFP reporter gene. The transfected B cells were cultured with genetically engineered fibroblastic BJ (feeder) cells expressing CD40L and secreting IL-21 (Figure 1A,F and Supplementary Figure S1A), which have been shown to strongly induce B-cell proliferation [20,21]. The B cells in expansion were subject to flow cytometric analysis for observation of CD19+GFP+ immortalized cell frequency changes. Cells that were CD19+GFP+ increased in frequency to around 30% of the culture at day 14, and to about 60% of the culture at day 21, after which the CD19+GFP+ cells were isolated via cell sorting technique (Figure 1B and Supplementary Figure S1B). We observed varied frequencies of CD19+GFP+ cells in B cells from different NDs after 14-day and 21-day expansion periods (Supplementary Figure S1C), indicating the impact of intrinsic qualities of B cells on cell immortalization. With expansion and activation signals from CD40L and IL-21 provided by the feeder cells, flow cytometric analysis of the immortalized B cells showed that they expressed CD19, HLA-ABC, and HLA-DR, and highly express the activation markers CD40, CD80, CD83, and CD86 (Figure 1C and Supplementary Figure S1D). To test the optimal cell quantity for B-cell immortalization, different quantities (respectively 0.1, 0.25 and 0.5 × 10^6^) of B cells isolated from PBMCs of two NDs were immortalized, with GFP and CD19 expression analyzed via flow cytometry at day 10. We observed a clearly improved retroviral transduction and GFP and CD19 expression with at least 0.5 × 10^6^ B cells as the input cell quantity (Figure 1E).

### 3.2. IBMAM: Antigen Presentation Efficiency Assessment with MITD/MITD Variants and Variable Epitope Arrangements

**Figure 2:**
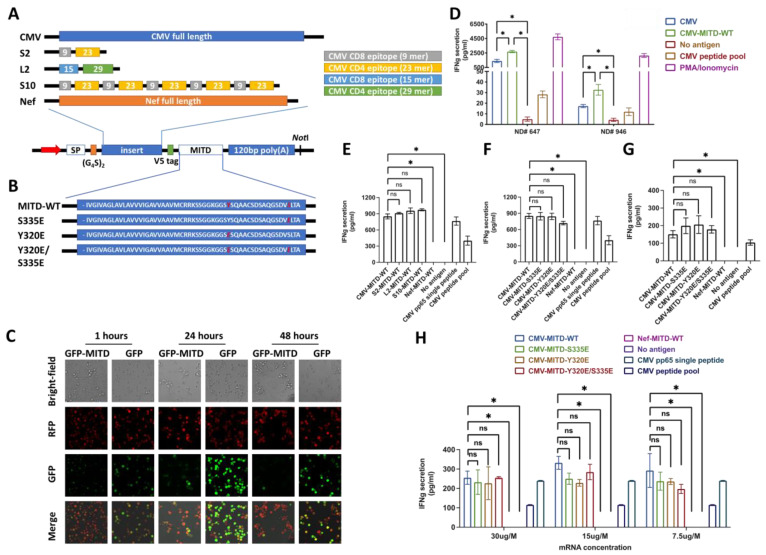
IBMAM: Antigen presentation efficiency assessment with MITD/MITD variants and variable epitope arrangements. (**A**,**B**) Schematic representation of the CMV-MITD vector template used for mRNA generation. Each construct contains the signal peptide (SP), MITD and 120 bp poly(A) tail. The pMRNA-120 bp-based plasmids were linearized with restriction enzyme NotI and used as templates for in vitro transcription (IVT). After purification, IVT was performed with the T7 polymerase using the mMESSAGE mMachine Ultra T7 kit (ThermoFisher). As shown in (**A**), five vector templates were generated, containing MITD-WT and respectively CMVpp65-full length gene (CMV-MITD-WT), Nef full length gene (Nef-MITD-WT), S2 sequence (S2-MITD-WT), L2 sequence (L2-MITD-WT) and S10 sequence (S10-MITD-WT). The S2 design combined the MHC-I and II epitopes for CMV pp65 with a disulfide linker ((G_4_S)_2_) in between. The short-10 (S10) sequence was modified to repeat the S2 gene design five times. The long-2 (L2) gene sequence adds three amino acids to the left and right side of the MHC-I and II epitopes, respectively, and then combines the modified MHC-I and II epitopes as described for the S2 gene sequence. As shown in (**B**), the other set of four CMV-MITD constructs were generated, containing either the CMVpp65-full length gene and respectively MITD-WT (pCMV-MITD-WT), the MITD-S335E (pCMV-MITD-S335E), the MITD-Y320E (pCMV-MITD-Y320E) or the MITD-Y320E/S335E (pCMV-MITD-Y320E/S335E) genes. The cDNA encoding MITD with a CMV pp65 epitope (pCMV-MITD-WT) was used as a template to generate point mutations at S335 and/or Y320. (**C**) Confocal imaging of mRNA expression. mRNA of GFP or GFP-MITD-WT was electroporated into immortalized B cells, and confocal imaging was conducted at 1, 24, 48 h post-mRNA transduction. (**D**) Detection of IFNg secretion from T cells isolated from different CMV+ NDs. CD4+ T cells and B cells were isolated from the PBMCs of CMV+ NDs (#946 and #647). The B cells were immortalized as described before. The CD4+ T cell were stimulated with IBMAM involving CMV-MITD-WT or CMV. IFNg secretion was detected after 24-h incubation. (**E**) Anti-CMV T-cell stimulation IFNg detection assay comparing the antigen presentation using CMVpp65 full length, S2, L2, and S10 when conjugated with MITD. The mRNA from the corresponding vector templates shown in (**A**) was generated and electroporated into Pt. 1 B cells, which were then used for anti-CMV CD8+ T-cell stimulation. Two negative controls included one group with no mRNA for B-cell electroporation, and the other with electroporation of mRNA from the vector containing Nef full length gene and MITD-WT. Two positive controls included the CMV pp65 single peptide and CMV peptide pool. IFNg was detected from each group after 48 h incubation. (**F**,**G**) Anti-CMV T-cell stimulation assay testing MITD-WT and MITD variants. The mRNA from the corresponding vector templates as mentioned in (**B**) was produced and electroporated into Pt. 1 B cells, which were subsequently co-incubated with either anti-CMV CD8+ (**F**) or anti-CMV CD4+ (**G**) T cells. The same negative and positive controls were used as previously described. IFNg was detected from each group after 48 h incubation. (**H**) The experiment described in (**F**) was further conducted at different mRNA concentrations, respectively at 7.5, 15, and 30 ug/10^6^ cells. See also Figures S2 and S3. * (*p* < 0.05).

Our ultimate goal is to develop a platform that can present unknown or natural epitopes to antigen-specific T cells for T-cell expansion. To achieve this, we utilized transfection with the full-length mRNA of antigen for naturally processed epitope presentation as it allows us to present epitopes that are not recognized. Our study only uses peptide epitopes as controls for the transfected mRNA. Recent studies have explored different strategies of improving antigen trafficking and presentation via linking the antigens to the endocytic-sorting motifs, which are the trafficking sequences of integral membrane proteins [5]. Among these motifs, MITD, when attached to the antigen, has been shown to strongly improve the presentation of both MHC class I and class II epitopes in human APCs [7]. To validate the assumption that MITD conjugation may assist in antigen presentation, we constructed a vector for RNA-encoding fusion proteins, in which the proteins of interest (insert) was ligated in between secretion signal peptide (SSP) and wild-type MITD (MITD-WT) sequences consisting of the transmembrane and cytoplasmic domain of the MHC-I molecule (Figure 2A). First, we tested the influence of MITD-WT via transfecting the immortalized B cell expressing RFP with mRNA encoding GFP or GFP-MITD-WT fusion proteins: confocal microscopy imaging was taken at 1, 24, and 48 h after mRNA electroporation. Our results clearly show that antigen expression occurs in a very fast manner after mRNA electroporation. While cells transfected with GFP alone displayed, as expected, a diffuse nuclear and cytoplasmic distribution, cells transfected with GFP-MITD exhibited green fluorescence accentuation of the cell membrane and inner cell organelle. These results indicate that our construct enabled GFP to enter endosomal compartments and can be used for efficient antigen presentation. Our results also validate the previous research showing that MITD guides the fused protein to the MHC-I/II Golgi apparatus for rapid decomposition and facilitates exposing of the peptide to the cell surface through MHC-I/II (Figure 2C and Supplementary Figure S2). While it has been widely acknowledged that MITD can increase antigen presentation efficacy when coupled to antigens, here we aimed to experimentally verify the effectiveness of MITD in enhancing antigen presentation in B cells to elicit the antigen-specific T-cell response. CD4+ T cells and B cells were isolated from PBMCs of CMV-seropositive NDs (ND #946 and #647), and B cells were immortalized as previously described. The immortalized B cells were transfected with mRNA encoding CMVpp65 peptide or peptide linked to MITD-WT, followed with incubation with autologous T cells. We observed an activation of CMVpp65-specific interferon-gamma (IFNg)-secreting CD4+ T cells with the CMV-MITD-WT mRNA, which was significantly higher than in absence of the MITD domain. Our findings confirm that MITD enhances antigen presentation compared to mRNA without MITD. Therefore, in our next experiments we will focus on using mRNA encoding the antigen fused with MITD. Our results suggest that the combination of the antigen with MITD in immortalized B cells is a highly effective method for stimulating and detecting antigen-specific T cells (Figure 2D).

Next, we aimed to investigate the epitope expression and antigen-specific T-cell activation via manipulating the epitope quantity and arrangement. The well-established CMVpp65 peptide sequence was used, which includes two core peptides that are respectively HLA-A*0201 restricted 9 mer (NLVPMVATV) for CMV-specific CD8+ T-cell epitope and HLA-DRB1*0701 restricted 23 mer (PLKMLNIPSINVHHYPSAAERKH) CMV-specific CD4+ T-cell epitope. We constructed a series of vector templates for RNA-encoding fusion CMV proteins, in which an array of CMV peptides were ligated in between SSP and MITD-WT sequence. While the short-2 (S2) sequence was designed to contain one HLA-A*0201 restricted 9 mer and one HLA-DRB1*0701 restricted 23 mer, long-2 (L2) sequence consists of one relatively longer peptide containing HLA-A*0201 restricted CMVpp65 epitope (15 mer: LARNLVPMVATVQGQ; underlined indicate core peptides) linked with another relatively longer peptide containing HLA-DRB1*0701 restricted CMVpp65 epitope (29 mer: YALPLKMLNIPSINVHHYPSAAERKHRHL; underlined indicate core peptides). Short-10 (S10) is the sequence containing five repeats of S2 sequence (Figure 2A, Supplementary Figure S3A–C, and Supplemental Table S1). HLA-matched B cells (HLA-A*0201) transfected with these RNA species or pulsed with synthetic CMVpp65 single peptide or peptide pools were used for in-vitro stimulation of commercially available HLA-A*0201 restricted anti-CMV CD8+ T cells (asteria comp.). We observed a substantial T-cell activation and cytokine release induced by B-cells processing mRNAs of CMVpp65-full-length-MITD-WT (CMV-MITD-WT), S2-MITD-WT, L2-MITD-WT and S10-MITD-WT. Moreover, we found that when conjugated with MITD, CMVpp65-full length exhibited satisfactory capability in stimulating T cells, and there was no significant difference detected among CMVpp65-full length, S2, L2 and S10 constructs. Our data suggests that in this system, using mRNA encoding a small number of key epitopes can have a similar effect as using mRNA encoding the full-length antigen in terms of stimulating antigen-specific T cells (Figure 2E). 

Previous studies have shown that phosphorylation on serine (Ser-335) and/or tyrosine (Tyr-320) in MITD plays a critical role in regulating class I MHC molecule’s sorting through the intracellular compartments [22]. Protein phosphorylation such as phosphor-Ser are phosphor-Tyr are very short-lived in cellular systems [23]. We hypothesize that mimicking phosphorylation of MITD can increase antigen presentation efficiency. We generated three phosphor-mimicking Tyr-to-Glu (Y320E), Ser-to-Glu (S335E), and Y320E/S335E mutations in MITD fused with CMVpp65 full-length sequences (Figure 2B and Figure S3D). The corresponding set of RNA species were electroporated into the HLA-matched B cells which were subsequently used for stimulation analysis of anti-CMV CD8+ T cells (Cellero Inc., Lowell, MA, USA) and anti-CMV CD4+ T cells isolated from CMV-seropositive NDs’ PBMC samples. Whereas B cells electroporated with CMVpp65-full length conjugated with four different MITD formats (i.e., CMV-MITD-WT, CMV-MITD-S335E, CMV-MITD-Y320E, and CMV-MITD-Y320E/S335E) [22], all exhibit a remarkable T-cell stimulation, MITD-WT shows a relatively comparable antigen-presentation ability as other MITD variants (Figure 2F,G). Due to the concern that the mRNA quantity utilized might have reached the maximal mRNA processing and epitope presentation ability of B cells and led to lack of difference among groups of MITD variants, varying amounts (30 ug, 15 ug, and 7.5 ug mRNA per 10^6^ B cells) of RNA mentioned above were used for B-cell processing and stimulation of anti-CMV CD8 T cells. Only at the RNA amount of 15 ug per 10^6^ B cells, we found a relatively higher T-cell response in using CMV conjugated to MITD-WT (i.e., CMV-MITD-WT), compared to CMV linked with other MITD modifications. At 30 ug and 7.5 ug per 10^6^ B cells RNA amounts, we did not detect any difference in antigen presentation ability between MITD-WT and any other MITD-variants groups (Figure 2H). 

### 3.3. Assessment of the Interaction between Antigen-Specific T Cells and IBMAM

**Figure 3:**
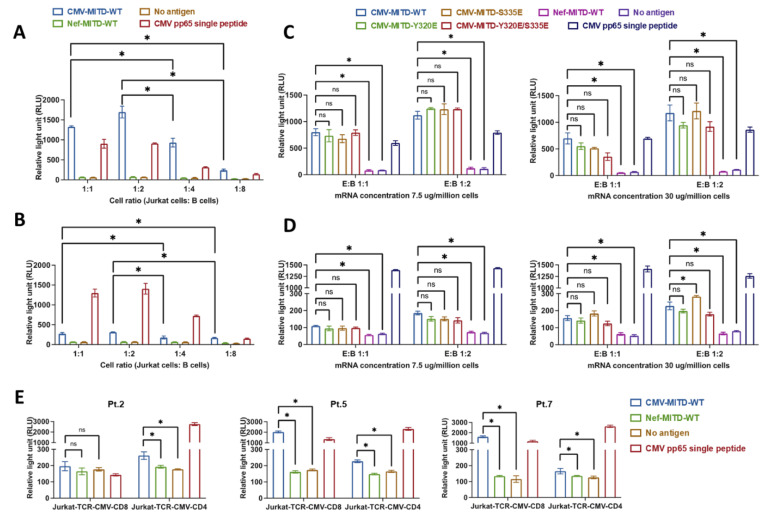
Assessment of the interaction between antigen-specific T cells and IBMAM. (**A**) Testing Jurkat:B cell ratios for optimal Jurkat (CD8+) cell activation. The CMV-MITD-WT mRNA was electroporated into Pt. 1 B cells (HLA-A*0201), which was used for stimulating Jurkat-CMV-TCR-CD8+. The stimulation with different ratios of Jurkat:B cells underwent 24 h, followed with the luminescence detection from stimulated Jurkat cells. (**B**) Testing Jurkat:B cell ratios for optimal Jurkat (CD4+) cell activation. The CMV-MITD-WT mRNA was electroporated into Pt. 5 B cells (HLA-DRB1*0701), which was used for stimulating Jurkat-CMV-TCR-CD4+. The stimulation with different ratios of Jurkat:B cells was for 24 h, followed with the luminescence detection from stimulated Jurkat cells. (**C**) Jurkat (CD8+) cell stimulation assay with Pt. 1 B cells using two Jurkat:B ratios. The mRNA from the corresponding vector templates as mentioned in Figure 2B was produced and electroporated into Pt. 1 B cells, which were subsequently co-incubated stimulating Jurkat-CMV-TCR-CD8+ cells. The experiment was conducted at mRNA concentrations respectively at 7.5 and 30 ug/10^6^ B cells and Jurkat: B cell ratios of 1:1 and 1:2. (**D**) Jurkat (CD4+) cell stimulation assay with Pt. 5 B cells using two Jurkat:B ratios. The mRNA from the corresponding vector templates as mentioned in Figure 2B was produced and electroporated into Pt. 5 B cells, which were subsequently co-incubated stimulating Jurkat-CMV-TCR-CD4+ cells. The experiment was conducted at mRNA concentrations respectively at 7.5 and 30 ug/10^6^ B cells and Jurkat: B cell ratios of 1:1 and 1:2. (**E**) Jurkat stimulation assay using B cells from lymphoma patients’ PBMC samples. B cells were isolated from PBMCs from Pt. 2, Pt. 5, and Pt. 7 and immortalized as previously described. The immortalized B cells were electroporated with the CMV-MITD-WT mRNA at 30 ug/10^6^ B cells, and cocultured with Jurkat-CMV-TCR-CD8+ and Jurkat-CMV-TCR-CD4+ at Jurkat:B cell ratio of 1:1 for 24 h before luminescence detection. In Figure 3B–E, negative controls included one group with no mRNA for B-cell electroporation, and the other with electroporation of mRNA from the vector containing Nef full length gene. Positive controls included the CMV pp65 single peptides and CMV peptide pool. See Figure S4. * (*p* < 0.05).

Next, we investigated the cell ratio of T:B cell, which is of critical importance for efficient induction of antigen-specific T-cell response. The TCRαβ-KO Jurkat cells (Promega) were used, in which the transient transfection of PCI mammalian vector encoding TCRαβ enables the concurrent expression of both TCR and CD3 (Supplementary Figure S4A,B). While these cells express a luciferase reporter driven by a TCR pathway-dependent promoter, activation of transgenic TCR-expressing cells by cognate peptide and MHC-expressing APCs results in potent TCR activation and promoter-mediated luminescence. The PCI mammalian vectors were constructed to express CMVpp65-specific TCRαβ with either CD8+ phenotype (HLA-A*0201 restricted; recognized region: aa495–503 [15,24]) or CD4+ phenotype (HLA-DRB1*0701 restricted; recognized region: aa337–359 [15,25]), and electroporated into the Jurkat cells, to produce Jurkat-CMV-TCR-CD8+ or Jurkat-CMV-TCR-CD4+ cells (Supplementary Figure S4C,D). We cocultured CMVpp65-full-length-MITD-WT (i.e., CMV-MITD-WT) mRNA-electroporated immortalized B cells with Jurkat T cells at Jurkat:B cell ratios of 1:1, 1:2, 1:4, and 1:8, and analyzed the Jurkat T-cell activation via luminescence detection. We found the Jurkat:B cell ratios of 1:1 and 1:2 were significantly more effective than 1:4 and 1:8 ratios, either when Jurkat-CMV-TCR-CD8+ (Figure 3A) or Jurkat-CMV-TCR-CD4+ (Figure 3B) was used. 

Using Jurkat:B cell ratios of 1:1 and 1:2, we further tested the usage of mRNA concentrations at 7.5 ug or 30 ug per 10^6^ immortalized B cells, which were respectively electroporated with CMVpp65-full length conjugated with four different MITD variant formats (i.e., CMV-MITD-WT, CMV-MITD-S335E, CMV-MITD-Y320E and CMV-MITD-Y320E/S335E). Again, the results did not show any significant difference between MITD-WT and other MITD variants in antigen presentation ability, and on the basis of this data, MITD-WT is now routinely used in our studies. In addition, with using CMV-MITD-WT mRNA, we did not detect any significant differences between the 7.5 ug and 30 ug per 10^6^ immortalized B cells, with respect to either Jurkat-CMV-TCR-CD8+ cell (Figure 3C) or Jurkat-CMV-TCR-CD4+ cell (Figure 3D) responses. These results support a mRNA concentration of 7.5–30 ug/10^6^ B cells as recommended for B-cell electroporation. 

On the basis of this data, we conclude the routine usage of MITD-WT for antigen linkage, mRNA concentration of 7.5–30 ug per 10^6^ immortalized B cells and Jurkat:B cell ratios of 1:1 or 1:2. Next we further tested whether these conditions can be widely applied. We used Jurkat TCRαβ-KO cells expressing CMVpp65-specific TCRs with either CD8+ phenotype (HLA-A*0201 restricted) or CD4+ phenotype (HLA-DRB1*0701 restricted) constructed as described above. B cells were isolated and immortalized from PBMC samples of three lymphoma patients, respectively Pt.2 (HLA-DRB1*0701), Pt.5 (HLA-A*0201 and HLA-DRB1*0701), and Pt.7 (HLA-A*0201 and HLA-DRB1*0701). B cells were electroporated with CMV-MITD-WT mRNA at 30 ug/10^6^ cells, and cocultured with either Jurkat-CMV-TCR-CD8+ cells or Jurkat-CMV-TCR-CD4+ cells at Jurkat:B cell ratio of 1:1. While Pt.5 and Pt.7 B cells were HLA-matched to both Jurkat-CMV-TCR-CD8+ cells or Jurkat-CMV-TCR-CD4+ cells, the results showed that the immortalized B cells processing CMV-MITD-WT mRNA could present epitopes effectively to both Jurkat-CMV-TCR-CD4+ and -CD8+ cells. While Pt.2 B cells were only HLA-DRB1*0701 matched to Jurkat-CMV-TCR-CD4+ cells but not Jurkat-CMV-TCR-CD8+ cells, only coculturing with Jurkat-CMV-TCR-CD4+ cells was able to induce significant Jurkat cell response (Figure 3E). 

### 3.4. General Schema of IBMAM Application in Antigen-Specific T-Cells Expansion

Following the characterization of immortalized B cells, MITD, and mRNA of candidate epitopes, which are the key components of IBMAM platform, we aimed to develop a general application schema of IBMAM platform to expand the low-frequency antigen-specific T cells in PBMCs which has been a common issue encountered by many research groups and for immunomonitoring in clinical trials of our DNA vaccines for lymphoma [26].

We decided to focus on using antigen-specific CD4+ T cells to test the capability of IBMAM platform, due to multiple reasons. First, neoantigen-specific CD4^+^ T-cell reactivity is common in human cancers [27,28]. Second, our group demonstrated MHC-II presentation and CD4+ T-cell recognition of the B-cell immunoglobulin in humans [29,30,31]. In addition, another group recently showed that lymphoma immunoglobulin neoantigens are presented abundantly by MHC-II for CD4+ T-cell recognition, but not MHC-I [32].

In this experiment, CD4+ T cells and B cells were isolated from the PBMCs of a number of different CMV-seropositive NDs. Immortalized B cells transfected with CMV-MITD-WT mRNA was cocultured with CD4+ T cells, followed by ELISA testing IFNg secretion. Our results showed that CMV-MITD-WT mRNA-transfected B cells induced antigen-specific T-cell activation in ND #673. No activation was observed in the negative controls (no antigen and Nef-MITD-WT mRNA) or with the B cells loaded with CMVpp65 peptide pool. However, we were not able to detect CMVpp65-specific CD4^+^ T cells in the other donor (ND #739) despite being CMV-seropositive ND.This may indicate that ND #739 had low frequency of CMV specific CD4+ T cells. It is noteworthy that we did not test the expansion of antigen-specific T cells from PBMCs of donors who are seronegative for CMV. This is because CMV is a widespread infection, being seronegative does not necessarily mean that there are no CMV-specific T cells. Furthermore, it is difficult to conclude whether the IBMAM platform is ineffective or if there are truly no CMV-specific T cells when using CMV seronegative PBMC samples and unable to expand CMV-specific T cells. However, using CMV seropositive PBMC samples will circumvent this issue.

**Figure 4:**
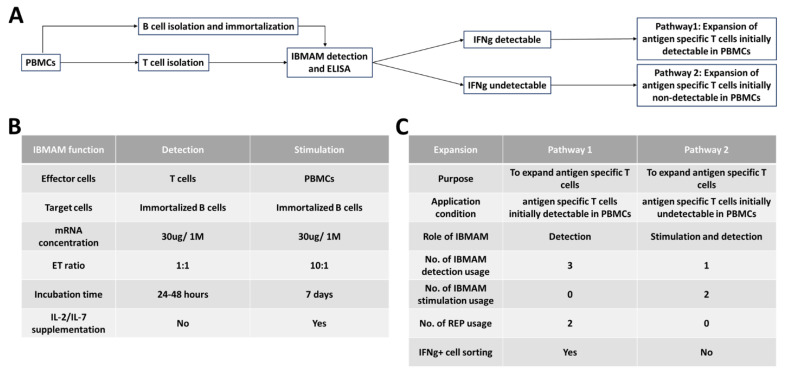
General schema of IBMAM application in antigen-specific T-cells expansion. (**A**) General schema representing the methodology of immunomonitoring and expansion of antigen-specific T cells from PBMCs. T cells are isolated from PBMCs and incubated with IBMAM system for IFNg+ antigen-specific T-cell detection. If IFNg can be detected, pathway 1 will be conducted for antigen-specific T-cell expansion. If IFNg is not able to be detected, pathway 2 will be used for antigen-specific T-cell IBMAM stimulation and expansion. (**B**) Comparison between two IBMAM system applications. IBMAM system can be used for detection of antigen-specific T cells or stimulation of PBMCs. The application conditions were illustrated in detail, including effector: target cell (E:T) ratio, incubation time and cytokine supplementation. E:T ratios represent T:B cell ratio in IBMAM detection and PBMC:B cell ratio in IBMAM stimulation. (**C**) Comparison between pathway 1 and 2. While use of pathway 1 or 2 depends on whether antigen-specific T cells can be detected initially from PBMCs, IBMAM system functions differently in these two pathways. The number of IBMAM detection and IBMAM stimulation usage, and number of REP usage, as well as cell sorting usage are compared in both pathways.

Under two conditions in which the IFNg/antigen-specific T cells can be detectable or undetectable, we developed two pathways using IBMAM for antigen-specific T-cell expansion (Figure 4). While the antigen-specific T-cell expansion serves as the goal, IBMAM possesses two key functions which are the detection of antigen-specific T cells and stimulation of PBMCs.

**Figure 5:**
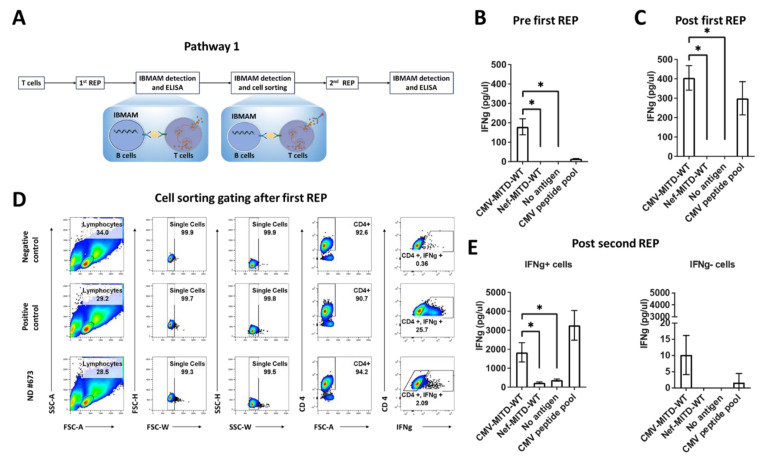
Pathway1: Expansion of antigen-specific T cells initially detectable in PBMCs using IBMAM. (**A**) General schema showing the experimental design for expansion of CMV-specific T cell. CD4+ T cells isolated from CMV+ ND PBMCs were expanded using REP. IFNg secretion from CMV-specific T cells were detected by co-culturing CD4+ T cells with IBMAM system and IFNg+/IFNg- CD4+ T cells were sorted out after application of IBMAM system. IFNg+/IFNg- CD4+ T cells were expanded via second REP and IFNg secretion was confirmed by IBMAM system. (**B**,**C**) IFNg detection using ELISA. IFNg concentration in culture supernatants after co-culturing IBMAM system and CD4+ T cells (**B**) or CD4+ T cells after first REP (**C**) was detected. (**D**) Cell sort gating of IFNg+/IFNg- CD4+ T cells from ND #673 using IFNg capture assay. CD4+ T cells were co-cultured with B cells with non-antigen or SEB served as negative and positive controls for gating IFNg+ cells. IFNg+/IFNg- CD4+ T cells were sorted after incubated with IBMAM system involving CMV-MITD. (**E**) IFNg detection assay. The sorted IFNg+/IFNg- CD4+ T cells were expanded using second REP, and IFNg secretion was confirmed via co-culturing IBMAM system and IFNg+/IFNg- CD4+ T cells. * (*p* < 0.05).

For IFNg+ antigen-specific T-cell expansion, if antigen-specific T cells are first detectable in PBMC samples using initial IBMAM detection, the T cells are extracted from initial PBMCs and a T-cell expansion using rapid expansion procedures (REP) is performed [33]. Expanded T cells are IBMAM detected for antigen-specificity and then sorted. A second REP is then performed, and double expanded T cells undergo IBMAM detection testing for antigen-specificity. This details pathway 1 of the IBMAM platform (Figure 5). 

**Figure 6:**
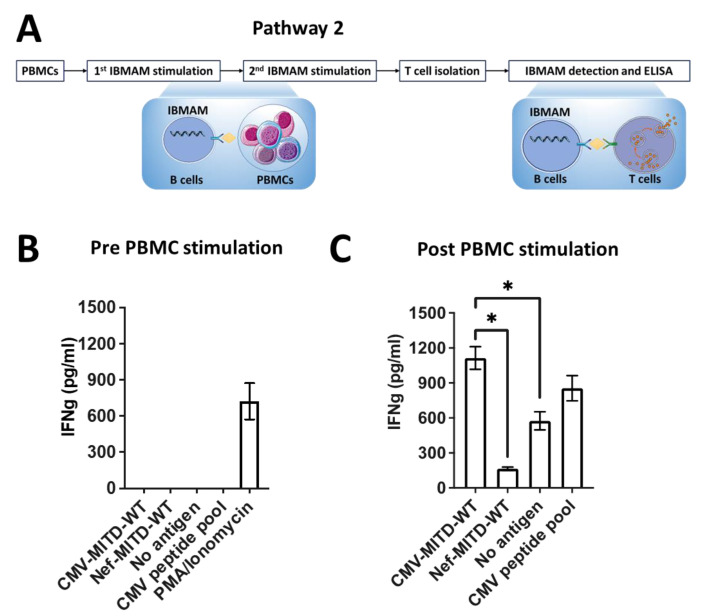
Pathway2: Expansion of antigen-specific T cells initially non-detectable in PBMCs using IBMAM. (**A**) General schema showing the experimental design for CMV-specific T-cell stimulation. PBMCs from CMV+ ND were stimulated twice with IBMAM before the CD4+ T-cell isolation. The isolated CD4+ T cells were incubated with IBMAM, followed with IFNg secretion detection using ELISA. (**B**) IFNg detection assay before PBMC and B-cell coculturing. IFNg concentration were detected via co-culturing IBMAM system and CD4+ T cells isolated from PBMCs. (**C**) IFNg detection assay after PBMC and B-cell coculturing. PBMCs were stimulated with IBMAM involving CMV-MITD-WT at B:PBMC ratio of 1:10 for twice, and CD4+ T cells were subsequently isolated from the stimulated PBMCs and co-incubated with IBMAM for IFNg detection. See Figure S5. * (*p* < 0.05).

If IFNg+ antigen-specific T cells are first undetectable from PBMC samples, we stimulate the PBMCs twice with IBMAM. T cells are then isolated from the stimulated samples and a final IBMAM detection of the isolated T cells is performed as a last check for antigen-specificity. This details pathway 2 of the IBMAM platform (Figure 6).

Next, we tested whether the proposed two pathways could be applied to successfully expand antigen-specific T cells from PBMC samples. 

### 3.5. Pathway1: Expansion of Antigen-Specific T Cells Initially Detectable in PBMCs Using IBMAM

In this study, we started with PBMC samples from CMV-seropositive ND #673; B cells were isolated from PBMCs and immortalized using the previously mentioned methodology, with activation marker expression analyzed via flow cytometric analysis. In here IBMAM platforms were used, respectively immortalized B cells processing CMV-MITD-WT mRNA (CMV-IBMAM) or immortalized B cells processing Nef-MITD-WT mRNA (Nef-IBMAM) as negative control of CMV-IBMAM. CD4+ T cells were isolated and cocultured with IBMAM or immortalized B cells loaded with no antigen or peptide pool controls. Our data shows that the CMV-IBMAM possesses effective capability in detecting antigen-specific T-cell response (Figure 5A,B).

Next, CD4+ T cells underwent the first REP expansion. The expanded cells were subsequently subjected to the IFNg secretion assay where CD4+ T cells were respectively incubated with the IBMAM or immortalized B cells loaded with CMVpp65 peptide pool or no antigen controls, or B cells with SEB. The IFNg ELISA result shows a remarkable amount of IFNg secretion detected using CMV-IBMAM platform, and there is nearly two-to-three-fold increase of IFNg secretion after first REP expansion (Figure 5C). While respectively 0.36% and 25.7% IFNg+ cells were observed in B cells plus no antigen stimulated and B cells plus SEB stimulated CD4+ T-cell culture, nearly 2.08% IFNg+ cells were detected in the CD4+ T cells after incubation with CMV-IBMAM platform; the IFNg+ and IFNg- cells were then sorted out and subjected to the second REP proliferation (immortalized B cells with SEB and immortalized B cells with no antigen were the respective positive and negative controls) (Figure 5D).

The expanded IFNg+ and IFNg- CD4+ T cells after the second REP were incubated with IBMAM or immortalized B cells loaded with CMVpp65 peptide pool, and the IFNg detection was performed via ELISA. Our data clearly shows that after the second REP, significantly higher secretion of IFNg can be detected from the IFNg+ cells using CMV-IBMAM (Figure 5E). Above all, through the CMV model, we show that the IBMAM is effective in expanding antigen-specific T cells which can initially be detectable in PBMCs. With the IBMAM, sufficient antigen-specific T cells can be obtained without loss of function, allowing further experimental use.

### 3.6. Pathway2: Expansion of Antigen-Specific T Cells Initially Undetectable in PBMCs Using IBMAM

To test the capability of IBMAM in facilitating the expansion of antigen-specific T cells which are initially undetectable in PBMCs, we first isolated B cells and CD4+ T cells from PBMCs of CMV-seropositive #739 ND. The B cells were immortalized with above-described protocol. The CD4+ T cells were subsequently incubated with CMV-IBMAM, Nef-IBMAM or immortalized B cells loaded with CMVpp65 peptide pool. Our results show that no IFNg was detected from CD4+ T cells from #739 ND (despite the patient being seropositive for CMV) (Figure 6A,B).

Next, PBMCs of #739 ND were stimulated twice with irradiated immortalized B cells processing CMV-MITD-WT mRNA (CMV-IBMAM), respectively on day 1 and day 8, and two ratios of PBMC:B cells were tested (1:1 and 10:1). On day 15, the CD4+ T cells were isolated from the stimulated PBMCs, which potentially contained the CMV antigen-specific T cells. The isolated CD4+ T cells were subsequently incubated with CMV-IBMAM, Nef-IBMAM, or immortalized B cells loaded with no antigen to test the CMV-specific T-cell expansion. Our results show that in comparison to a PBMC:B cell ratio at 1:1, incubation at PBMC:B cell ratio at 10:1 led to a significantly larger expansion of CMV-specific CD4+ T cells, which were able to mount a remarkably higher IFNg secretion (Supplementary Figure S5). 

When the isolated CD4+ T cells from the group of PBMC:B cell ratio at 10:1 were incubated with CMV-IBMAM, Nef-IBMAM, or B cells loaded with no antigen or CMVpp65 peptide pool, IFNg secretion from incubation with CMV-IBMAM was around eight-fold higher than that from incubation with Nef-IBMAM (Figure 6C). Comparing the CD4+ T-cell response (IFNg secretion) before and after two rounds of PBMC stimulation, our results clearly support an antigen-specific T-cell expansion effect using IBMAM platform. 

### 3.7. IBMAM Platform Application to TCR Validation

The expansion of antigen-specific T cells (achieved using the above-described pathway 1 and pathway 2), the TCR repertoire, which represent the “molecular tags” of T cells, have been widely studied to monitor the T cell clonality and diversity, specifically in the context of tumor immunology. While bulk or single-cell RNA sequencing has been able to successfully provide TCR profiling information, downstream biological experiments are required to confirm and discover truly reactive paired TCRs. 

We hypothesized that the IBMAM platform can be utilized for tumor specific TCR validation, but first we wanted to display proof of principle with known paired TCRs that recognize tumor antigens. 

To test the capability of IBMAM in TCR validation, we first selected two panels of TCRs which respectively recognize the New York esophageal squamous cell carcinoma 1 (NY-ESO-1) antigen and EBV latent membrane protein-2A (LMP2A), representing tumor and viral antigens. The first panel included paired TCRs, namely TCR-NYESO-KFJ05, TCR-NYESO-KFJ15 and TCR-NYESO-KFJ37, which have been characterized to recognize NY-ESO-1_60–72_-HLA-B*0702 [34]. The second panel comprised TCR-LMP2A-C1 and TCR-LMP2A-C2, which have been shown to react with LMP2A_484–493_-HLA-A*0101 [35]. The PCI mammalian vectors were constructed to express the above TCRs, and electroporated to TCRαβ-KO Jurkat cells for TCR expression. The mRNA of NY-ESO-1-MITD-WT and LMP2A-MITD-WT were produced as previously described (Supplementary Figure S6). 

**Figure 7:**
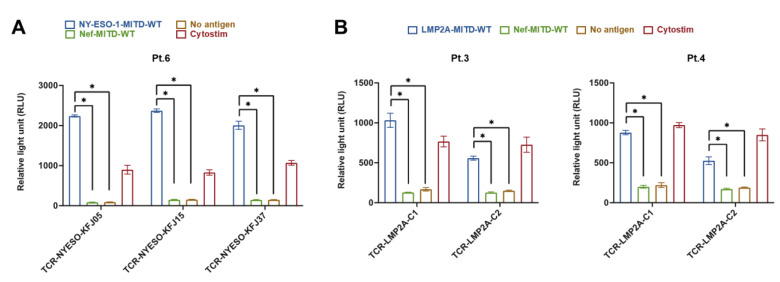
IBMAM platform application to TCR validation. (**A**) Jurkat stimulation assay testing the TCRs recognizing NY-ESO-1_60-72_. B cells were isolated from PBMCs from Pt. 6 and immortalized as previously described. The immortalized B cells were electroporated with the NY-ESO-1-MITD-WT mRNA at 30 ug/10^6^ B cells and cocultured respectively with Jurkat-TCR-NYESO-KFJ05, Jurkat-TCR-NYESO-KFJ15, and Jurkat-TCR-NYESO-KFJ37 at Jurkat:B cell ratio of 1:1 for 24 h before luminescence detection. (**B**) Jurkat stimulation assay testing the TCRs recognizing LMP2A_484–493_. B cells were isolated from PBMCs from Pt. 3 and Pt. 4 and immortalized as previously described. The immortalized B cells were electroporated with the LMP2A-MITD-WT mRNA at 30 ug/10^6^ B cells, and cocultured respectively with Jurkat-TCR-LMP2A-C1 and Jurkat-TCR-LMP2A-C2 at Jurkat:B cell ratio of 1:1 for 24 h before luminescence detection. In Figure 7A,B, negative controls included one group with no mRNA for B-cell electroporation, and the other with electroporation of mRNA from the vector containing Nef full length gene. Positive control included the incubation of Jurkat and B cells with cytostim. See Figure S6. * (*p* < 0.05).

For testing the TCRs recognizing NY-ESO-1_60–72_, the Jurkat cells expressing the TCRs (respectively Jurkat-TCR-NYESO-KFJ05, Jurkat-TCR-NYESO-KFJ15, and Jurkat-TCR-NYESO-KFJ37) were generated. Two IBMAM stimulations with the HLA-matched Pt. 6 B cells were utilized, respectively the B cells processing NY-ESO-1-MITD-WT mRNA (NY-ESO-1 IBMAM) or B cells processing Nef-MITD-WT mRNA (Nef-IBMAM) as negative control. The positive control group is the incubation of Jurkat cells and B cells with the addition of cytostim, which acts as a superantigen and causes activation of T cells by crosslinking TCR to MHC molecule of antigen-presenting cell. Our data shows that in comparison to Nef-IBMAM or B cells loaded with no antigen, incubation with NY-ESO-1 IBMAM led to a significantly stronger activation of Jurkat-TCR-NYESO-KFJ05, Jurkat-TCR-NYESO-KFJ15, and Jurkat-TCR-NYESO-KFJ37, confirming that our IBMAM platform can successfully present the epitope to these three Jurkat-expressing TCRs with functional reactivity against the NYESO-1 antigen (Figure 7A).

In the same manner described above, Jurkat TCRs recognizing LMP2A_484–493_ epitope were stimulated with IBMAMs (HLA-matched Pt. 3 and Pt. 4 B cells processing the mRNA of LMP2A-MITD-WT or Nef-MITD-WT), or B cells loaded with no antigen. The data clearly indicates the high specificity of TCR-LMP2A-C1 and TCR-LMP2A-C2 in recognizing the LMP2A epitopes (Figure 7B). Taken together, our results provide compelling evidence that IBMAM is an effective tool in validating the TCR reactivity, which can be applied in tumor specific high-throughput TCR screening and discovery from the patient paired TCR repertoire. 

## 4. Discussion

Here we have described a unique platform, in which we combined the immortalized B cells and mRNA of antigen conjugated with MITD molecule for increased antigen presentation. A stable autologous B-cell line was obtained by transducing B cells with BCL-6 and BCL-XL genes in the presence of IL-21 and CD-40L. These B cells highly expressed activation markers such as CD40, CD80, CD83, and CD86. Although the B cells were able to proliferate stably with feeder cells expressing CD40L and IL-21, we observed that prolonged proliferation might lead to a gradual decline of activation marker expression, which might be due to the effect of overactivation and loss of sensitization and/or the prolonged intracellular retention of retrovirus [10,36]. We further explored and optimized the whole B-cell immortalization schema in this method. Because limited patient sample is a significant complication for the study of antigen-specific T cells, we first investigated the minimum number of B cells needed for immortalization for future studies. Our study suggests that 0.5 × 10^6^ cells are the lowest number of cells required for B-cell immortalization. Previous studies have often involved DCs as effective antigen presenting cells transfected with mRNA encoding candidate epitopes for antigen presentation. Overall, the comparison between the potency of DCs and immortalized B cells as efficient and effective antigen presenting cells remains to be further explored. Whereas the usage of DCs involves isolation of immature DCs from PBMCs each time, induction of differentiation into mature DCs, and supplementation of CD14 and GM-CSF for cell growth, immortalized B cells appear as an unlimited “ready-to-use” resource with no need for induction for maturation [8]. 

The trafficking sequences of cytoplasmic, endosomal, or lysosomal proteins that reside in MHC-antigen processing compartments, such as invariant chain, LAMP, and dendritic cell (DC)-LAMP have been studied and linked to the antigens of interest to optimize MHC presentation. The fundamental molecular principle behind fusing antigens to endocytic sorting motifs to increase antigen presentation such as MITD is that the cytoplasmic tails of these proteins contain the conserved tyrosine- or leucine-based signals, which can interact with adaptor protein complexes and mediate translocation into defined subcellular compartments [37]. Such trafficking signals attaching to the interested antigens has been shown to improve the stimulation of antigen-specific T cells [6]. Invariant chain and LAMP proteins’ fusion of candidate antigens has led to more efficient class II-restricted antigen processing [38]. Our study clearly shows that CMV pp65 conjugated with MITD induced responses from both anti-CMV CD8+ and CD4+ T cells, validating MITD as a powerful tool for elevating the efficiency of both class-I and class-II restricted antigen processing. Conjugation with MITD that can facilitate the antigen processing and presentation is also visually evidenced by the confocal imaging of B cells processing GFP conjugated with MITD in our data. 

While our data using CMVpp65 minigenes show the efficacy of MITD in improving the antigen presentation and stimulation of antigen-specific T cells, it is of great interest to explore the optimal minigene construction for epitope presentation. Previous research involving DCs with mRNA minigene electroporation has shown that the neoantigen encoded by a minigene must be correctly cleaved and efficiently presented on the cell surface in conjunction with HLA class I molecules to elicit CD8+ T-cell responses [39]. Studies also suggest that in most cases, the order and arrangement of epitopes in minigenes is not of major importance for efficient presentation and discovery of individual neoepitopes [8]. However, few studies have ever explored the quantity of epitopes contained in mRNA for antigen presentation. In our study, various combinations of CD4+ and CD8+ T-cell recognized CMV epitopes (S2, L2, and S10 sequences) are reported to induce no significant differences in antigen-specific T-cell responses. Furthermore, we show that quantitatively increasing epitopic expression efficiency are ineffective in enhancing antigen presentation.

Early studies report that class I MHC molecules are phosphorylated on serine and tyrosine which occurs in a highly conserved region of the cytoplasmic domain [40,41], and class I MHC phosphorylation plays a role in regulating intracellular sorting through the intracellular compartments [42,43]. In Basha et al., the point mutation of tyrosine in the highly conserved sequence of class I MHC molecule gene leads to the failure of MHC-I molecules to gain access to peptide-loading sites due to aberrant intracellular trafficking [6]. While phosphorylation is essential for MHC molecule intracellular routing, it would be interesting to compare MITD-WT and MITD with tyrosine and/or serine phosphorylation in antigen presentation. Our results involving three MITD variants with serine/tyrosine phosphorylation show no significant difference from MITD-WT regarding the antigen presentation, supporting MITD-WT already as the optimal candidate for antigen presentation. While the underlying mechanism remains unknown and desires further exploration, some other means of MITD phosphorylation could be tested to improve antigen presentation further. For instance, in Guild BC et al., HLA-A2 and -B7 antigens could be phosphorylated by the 60,000-dalton phosphoprotein pp60v-src encoded by the Rous sarcoma virus [44]. 

The ratio between antigen presenting cells and antigen-specific T cells is essential for efficient T-cell reaction and detection. For instance, in Ali M et al., T:DC cell ratios of 1:1, 2:1, 4:1 and 8:1 were evaluated, and 2:1 and 4:1 T:DC cell ratios were found as the optimal ratios for T-cell stimulation [8]. While few studies have applied immortalized B cells as the antigen-presenting cells, in this study we test an array of Jurkat:B cell ratios and show that 1:1 and 1:2 Jurkat:B cell ratios serve as the optimal ratios for antigen presentation, which will be used in our future studies. Interestingly, the application of Jurkat:B cell ratios of 1:4 and 1:8 which involves increased input of B cells leads to a decline in Jurkat cell stimulation and activation. This is likely due to the exhaustion of Jurkat cells responding to a sustained overstimulation from antigen presenting cells, which is similar to the underlying mechanism of T-cell exhaustion where persistent antigen presentation causes continuous up-regulation of inhibitory receptors that facilitate delivery of negative signals [45]. Regarding the incubation of PBMCs with IBMAM for stimulation and expansion of the antigen-specific T cells, we show that using PBMC:B cell ratio of 10:1 leads to detectable expansion of antigen-specific T cells compared to using PBMC:B cell ratio of 1:1. This is reasonable due to the initial low frequency of T cells in PBMCs; an high cell number of PBMCs is required. Based on these observations, we report that cell ratios should be tested when IBMAM platform is used with different effector cells. 

Although the discovery of tumor-specific TCRs within a small volume of patient samples remains a significant technical challenge, we believe that the IBMAM platform can provide the solution. Additionally, the recent advancements in high-throughput single-cell sequencing allow for the possibility of single cell TCR sequencing of IBMAM stimulated and expanded antigen specific T cells. These TCRs can be then synthesized and transfected into the TCRαβ-KO Jurkat cell line, followed by tumor antigen recognition testing. Currently, we are using the IBMAM platform to discover and verify Idiotype-specific TCRs in the blood of patients treated with Idiotype DNA vaccines against lymphoplasmacytic lymphoma patients [26].

### Limitation of the Study

In our study, we show that the three MITD variants with serine/tyrosine phosphorylation (Y320E, S335E, and Y320E/S335E mutations) has no significant difference from MITD-WT in terms of antigen presentation, therefore, we now routinely use MITD-WT in all our studies. However, we have no direct evidence to show whether MITD phosphorylation is a feasible means in enhancing antigen presentation or if the mimicking phosphorylation that we designed is truly effective. In addition, recent studies have shown that minigene transfectants have lower reactivity compared to peptides and have limitations in eliciting reactivities [46]. The mechanism behind the inefficiency of class II processing and presentation of epitopes derived from intracellular proteins is not yet understood and requires further investigation.

## 5. Conclusions

In summary, our results provide an important platform (IBMAM) for the rapid ex vivo expansion of antigen specific T cells from low frequency circulating antigen-specific T cells in PBMC. Two pathways have been developed using IBMAM for antigen-specific T-cells expansion under two conditions in which antigen-specific T cells can be detectable or undetectable. Moreover, IBMAM has been shown to successfully validate viral and tumor specific TCRs, enabling its usage in TCR screening and discovery. Overall, the IBMAM platform is effective as a tool for immunomonitoring, antigen-specific T-cell identification and expansion, and antigen-specific TCRs discovery in extremely limited patient samples.

## Data Availability

The data that support the findings of this study are available on request from the corresponding author. The data are not publicly available due to privacy or ethical restrictions.

## Supplementary Materials

The following supporting information can be downloaded at: https://www.mdpi.com/article/10.3390/biomedicines11030796/s1, Figure S1: Characterization of B cell immortalization; Figure S2: Confocal imaging of immortalized B cells processing GFP-MITD-WT mRNA; Figure S3: Production of mRNA of CMV gene conjugated with MITD; Figure S4:Jurkat cell activation bioassay; Figure S5: Optimization of B cell: PBMC quantity ratios for PBMC stimulation; Figure S6: TCR expression on Jurkat cells and mRNA production. Table S1: HLA haplotypes from lymphoma patients. Table S2: Synthetic genes used in this study.
