# Supplementary material for: Immortalized B Cells Transfected with mRNA of Antigen Fused to MITD (IBMAM): An Effective Tool for Antigen-Specific T-Cell Expansion and TCR Validation
Source: Biomedicines. 2023 Mar 6;11(3):796. doi: 10.3390/biomedicines11030796 (PMC10045729; doi:10.3390/biomedicines11030796)
Supplement: Supplementary file 1 [file biomedicines-11-00796-s001.zip › biomedicines-2250000-supplementary.pdf]

Supplemental Table S1. HLA haplotypes from lymphoma patients

	HLA class I			HLA class II		
**Patient ID**	A	B	C	DRB1	DQB1	DPB1
**Pt.1**	02:01<br>03:01	18:01<br>40:01	03:04<br>07:01	01:01<br>04:01	03:02<br>05:01	02:01<br>04:01
**Pt.2**	01:01<br>30:01	08:01<br>13:02	06:02<br>07:01	03:01<br>07:01	02:01<br>02:02	01:01<br>17:01
**Pt.3**	01:01	08:01<br>37:01	06:02<br>07:01	03:01<br>15:01	02:01<br>06:02	02:01<br>04:01
**Pt.4**	01:01	08:01	07:01	03:01<br>15:01	02:01<br>06:02	02:01<br>14:01
**Pt.5**	02:01<br>32:01	15:01<br>51:01	03:03<br>14:02	01:01<br>07:01	03:03<br>05:01	04:01
**Pt.6**	03:01<br>11:01	07:02<br>40:01	03:04<br>07:02	13:02<br>15:01	06:02<br>06:04	03:01<br>04:01
**Pt.7**	02:01<br>24:02	40:01<br>55:01	03:03<br>03:04	07:01<br>15:01	02:02<br>06:02	04:01<br>04:02

Supplemental Table S2. Synthetic genes used in this study

Gene name	Synthetic DNA sequence	Information amino acid sequence
S2	**AGA TCT** AAC CTG GTA CCA ATG GTA GCT ACA GTG *GGT GGT GGT GGT TCT* CCG CTC<br>AAG ATG TTG AAT ATT CCG AGC ATT AAC GTC CAC CAT TAC CCA TCT GCT GCG GAG<br>AGA AAA CAT **CTC GAG**<br><br>Linker	**RS**NLVPMVATV*GGGGS*PLKMLNIPSINVHHY<br>PSAAERKH**LE**
S10	**AGA TCT** AAC CTG GTA CCA ATG GTA GCT ACA GTG *GGT GGT GGT GGT TCT* CCG CTC<br>AAG ATG TTG AAT ATT CCG AGC ATT AAC GTC CAC CAT TAC CCA TCT GCT GCG GAG<br>AGA AAA CAT *GGT GGT GGT GGT TCT* AAC CTG GTA CCA ATG GTA GCT ACA GTG *GGT*<br>*GGT GGT GGT TCT* CCG CTC AAG ATG TTG AAT ATT CCG AGC ATT AAC GTC CAC CAT<br>TAC CCA TCT GCT GCG GAG AGA AAA CAT *GGT GGT GGT GGT TCT* AAC CTG GTA CCA<br>ATG GTA GCT ACA GTG *GGT GGT GGT GGT TCT* CCG CTC AAG ATG TTG AAT ATT CCG<br>AGC ATT AAC GTC CAC CAT TAC CCA TCT GCT GCG GAG AGA AAA CAT *GGT GGT GGT*<br>*GGT TCT* AAC CTG GTA CCA ATG GTA GCT ACA GTG *GGT GGT GGT GGT TCT* CCG CTC<br>AAG ATG TTG AAT ATT CCG AGC ATT AAC GTC CAC CAT TAC CCA TCT GCT GCG GAG<br>AGA AAA CAT *GGT GGT GGT GGT TCT* AAC CTG GTA CCA ATG GTA GCT ACA GTG *GGT*<br>*GGT GGT GGT TCT* CCG CTC AAG ATG TTG AAT ATT CCG AGC ATT AAC GTC CAC CAT<br>TAC CCA TCT GCT GCG GAG AGA AAA CAT **CTC GAG**<br><br>Linker	**RS**NLVPMVATV*GGGGS*PLKMLNIPSINVHHY<br>PSAAERKH*GGGGS*NLVPMVATV*GGGGS*PLKM<br>LNIPSINVHHYPSAAERKH*GGGGS*NLVPMVA<br>TV*GGGGS*PLKMLNIPSINVHHYPSAAERKH*G*<br>*GGGS*NLVPMVATV*GGGGS*PLKMLNIPSINVH<br>HYPSAAERKHGGGGSNLVPMVATV*GGGGS*PL<br>KMLNIPSINVHHYPSAAERKH**LE**
L2	**AGA TCT** CTC GCC CGG AAC CTG GTC CCC ATG GTC GCC ACC GTG CAG GGG CAA *GGT*<br>*GGT GGT GGT TCT* TAC GCC CTG CCC CTG AAG ATG CTG AAC ATC CCA TCC ATC AAC<br>GTG CAC CAC TAT CCT TCC GCC GCC GAA CGG AAG CAT CGG CAC CTC **CTC GAG**	**RS**LARNLVPMVATVQGQGGGGSYALPLKMLN<br>IPSINVHHYPSAAERKHRHL**LE**
MITD-WT	*CTG CAT* ATC GTG GGC ATT GTT GCT GGC CTG GCT GTC CTA GCA GTT GTG GTC ATC<br>GGA GCT GTG GTC GCT GCT GTG ATG TGT AGG AGG AAG AGC TCA GGT GGA AAA GGA<br>GGG AGC **TAC** TCT CAG GCT GCG TGC AGC GAC AGT GCC CAG GGC TCT GAT GTG **TCT**<br>CTC ACA GCT TGA *GGA TCC*	*LQ*IVGIVAGLAVLAVVVIGAVVAAVMCRRKS<br>SGGKGGS**Y**SQAACSDSAQGSDV**S**LTA
MITD-Y320E	*CTG CAT* ATC GTG GGC ATT GTT GCT GGC CTG GCT GTC CTA GCA GTT GTG GTC ATC<br>GGA GCT GTG GTC GCT GCT GTG ATG TGT AGG AGG AAG AGC TCA GGT GGA AAA GGA<br>GGG AGC **GAG** TCT CAG GCT GCG TGC AGC GAC AGT GCC CAG GGC TCT GAT GTG **TCT**<br>CTC ACA GCT TGA *GGA TCC*	*LQ*IVGIVAGLAVLAVVVIGAVVAAVMCRRKS<br>SGGKGGS**E**SQAACSDSAQGSDV**S**LTA

MITD-S335E	*CTG CAT* ATC GTG GGC ATT GTT GCT GGC CTG GCT GTC CTA GCA GTT GTG GTC ATC<br>GGA GCT GTG GTC GCT GCT GTG ATG TGT AGG AGG AAG AGC TCA GGT GGA AAA GGA<br>GGG AGC **TAC** TCT CAG GCT GCG TGC AGC GAC AGT GCC CAG GGC TCT GAT GTG **GAG**<br>CTC ACA GCT TGA *GGA TCC*	*LQ*IVGIVAGLAVLAVVVIGAVVAAVMCRRKS<br>SGGKGGS**Y**SQAACSDSAQGSDV**E**LTA
MITD-Y320E/S335E	*CTG CAT* ATC GTG GGC ATT GTT GCT GGC CTG GCT GTC CTA GCA GTT GTG GTC ATC<br>GGA GCT GTG GTC GCT GCT GTG ATG TGT AGG AGG AAG AGC TCA GGT GGA AAA GGA<br>GGG AGC **GAG** TCT CAG GCT GCG TGC AGC GAC AGT GCC CAG GGC TCT GAT GTG **GAG**<br>CTC ACA GCT TGA *GGA TCC*	*LQ*IVGIVAGLAVLAVVVIGAVVAAVMCRRKS<br>SGGKGGS**E**SQAACSDSAQGSDV**E**LTA
NY-ESO-1	atgcaggccgaaggccggggcacaggggttcgacgggcgatgctgatggcccaggaggccctggcattcc<br>tgatggcccaggggcaatgctggcggcccaggagaggcgggtgccacgggcggcagaggtccccggggcg<br>caggggcagcaagggcctcggggccgggaggaggcgccccgcggggtccgcatggcggcgcggcttcaggg<br>ctgaatggatgctgcagatgcggggccaggggccggagagccgcctgcttgagttctacctcgccatgcc<br>tttcgcgacacccatggaagcagagctggcccgcaggagcctggcccaggatgccccaccgcttcccgtgc<br>caggggtgcttctgaaggagttcactgtgtccggcaacatactgactatccgactgactgctgcagaccac<br>cgccaactgcagctctccatcagctcctgtctccagcagctttccctgttgatgtggatcacgcagtgctt<br>tctgcccgtgtttttggctcagcctccctcagggcagaggcgc	MQAEGRGTGGSTGDADGPGGPGIPDGPGGNA<br>GGPGEAGATGGRGPRGAGAARASGPGGGAPR<br>GPHGGAASGLNGCCRCGARGPESRLLEFYLA<br>MPFATPMEAELARRSLAQDAPPLPVPGVLLK<br>EFTVSGNILTIRLTAADHRQLQLSISSCLQQ<br>LSLLMWITQCFLPVFLAQPPSGQRR
LMP2A	accgcaggattcctgatctttctgattggcttcgcgcttttcggggtaattaggtgctgtagatattgttg<br>ttattattgtctcaccctcgaatccgaagagcggcctcctacgccttaccggaatactgtaacggctggat<br>tccttatattccttatcggattcgctctgtttggggtcattaggtgctgccggtactgttgttactactgc<br>cttacgctggagtcagaggaacgccccctacaccatacaggaataccgtg	TAGFLIFLIGFALFGVIRCCRYCCYYCLTLE<br>SEERPPTPYRNTVTAGFLIFLIGFALFGVIR<br>CCRYCCYYCLTLESEERPPTPYRNTV
TCR CD8-CMV#8	**GAATTC**AGCGGCCGCACC**ATGGGCACGAGGCTCTTCTTCTATGTGGCCCTTTGTCTGCTGTGGGCAGGACACA**<br>**GGGATGCTGAAATCACCCAGAGCCCAAGACACAAGATCACAGAGACAGGAAGGCAGGTGACCTTGGCGTGT**<br>**CACCAGACTTGGAACCACAACAATATGTTCTGGTATCGACAAGACCTGGGACATGGGCTGAGGCTGATCCA**<br>**TTACTCATATGGTGTTCAAGACACTAACAAAGGAGAAGTCTCAGATGGCTACAGTGTCTCTAGATCAAACA**<br>**CAGAGGACCTCCCCCTCACTCTGGAGTCTGCTGCCTCCTCCCAGACATCTGTATATTTCTGCGCCAGCAGC**<br>**GACCCCACCGAGGAGAAGCTGTTCTTCGGCAGCGGCACCCAGCTGAGCGTGCTGGAGGAC***CTGAACAAGGT*<br>*GTTCCCACCCGAGGTCGCTGTGTTTGAGCCATCAGAAGCAGAGATCTCCCACACCCAAAAGGCCACACTGG*<br>*TGTGCCTGGCCACAGGCTTCTTCCCCGACCACGTGGAGCTGAGCTGGTGGGTGAATGGGAAGGAGGTGCAC*<br>*AGTGGGGTCAGCACGGACCCGCAGCCCCTCAAGGAGCAGCCCGCCCTCAATGACTCCAGATACTGCCTGAG*<br>*CAGCCGCCTGAGGGTTTCGGCCACCTTCTGGCAGAACCCCCGCAACCACTTCCGCTGTCAAGTCCAGTTCT*<br>*ACGGGCTCTCGGAGAATGACGAGTGGACCCAGGATAGGGCCAAACCCGTCACCCAGATCGTCAGCGCCGAG*<br>*GCCTGGGGTAGAGCAGACTGTGGCTTTACCTCGGTGTCCTACCAGCAAGGGGTCCTGTCTGCCACCATCCT*<br>*CTATGAGATCCTGCTAGGGAAGGCCACCCTGTATGCTGTGCTGGTCAGCGCCCTTGTGTTGATGGCCATGG*<br>*TCAAGAGAAAGGATTTC*aggaggaggaggagcggcagtggaGTGAAACAGACTTTGAATTTTGACCTTCTC<br>AAGTTGGCGGGAGACGTGGAGTCCAACCCAGGGCCC**ATGAAGAGGATATTGGGAGCTCTGCTGGGGCTCTT**<br>**GAGTGCCCAGGTTTGCTGTGTGAGAGGAATACAAGTGGAGCAGAGTCCTCCAGACCTGATTCTCCAGGAGG**<br>**GAGCCAATTCCACGCTGCGGTGCAATTTTTCTGACTCTGTGAACAATTTGCAGTGGTTTCATCAAAACCCT**<br>**TGGGGACAGCTCATCAACCTGTTTTACATTCCCTCAGGGACAAAACAGAATGGAAGATTAAGCGCCACGAC**<br>**TGTCGCTACGGAACGCTACAGCTTATTGTACATTTCCTCTTCCCAGACCACAGACTCAGGCGTTTATTTCT**<br>**GCGCCGTGGTGAGGTGGGAGACCAGCGGCAGCAGGCTGACCTTCGGCGAGGGCACCCAGCTGACCGTGAAC**<br>**CCCGACATCCAGAACCCCGACCCTGCC***GTGTACCAGCTGAGAGACTCTAAATCCAGTGACAAGTCTGTCTG*	**MGTRLFFYVALCLLWAGHRDAEITQSPRHKI**<br>**TETGRQVTLACHQTWNHNNMFWYRQDLGHGL**<br>**RLIHYSYGVQDTNKGEVSDGYSVSRSNTEDL**<br>**PLTLESAASSQTSVYFCASSDPTEEKLFFGS**<br>**GTQLSVLED***LNKVFPPEVAVFEPSEAEISHT*<br>*QKATLVCLATGFFPDHVELSWWVNGKEVHSG*<br>*VSTDPQPLKEQPALNDSRYCLSSRLRVSATF*<br>*WQNPRNHFRCQVQFYGLSENDEWTQDRAKPV*<br>*TQIVSAEAWGRADCGFTSVSYQQGVLSATIL*<br>*YEILLGKATLYAVLVSALVLMAMVKRKDF*RR<br>RRSGSGVKQTLNFDLLKLAGDVESNPGP**MKR**<br>**ILGALLGLLSAQVCCVRGIQVEQSPPDLILQ**<br>**EGANSTLRCNFSDSVNNLQWFHQNPWGQLIN**<br>**LFYIPSGTKQNGRLSATTVATERYSLLYISS**<br>**SQTTDSGVYFCAVVRWETSGSRLTFGEGTQL**<br>**TVNPDIQNPDPA***VYQLRDSKSSDKSVCLFTD*<br>*FDSQTNVSQSKDSDVYITDKTVLDMRSMDFK*<br>*SNSAVAWSNKSDFACANAFNNSIIPEDTFFP*<br>*SPESSCDVKLVEKSFETDTNLNFQNLSVIGF*<br>*RILLLKVAGFNLLMTLRLWSS-*

	CCTATTCACCGATTTTGATTCTCAAACAAATGTGTCACAAAGTAAGGATTCTGATGTGTATATCACAGACA<br>*AAACTGTGCTAGACATGAGGTCTATGGACTTCAAGAGCAACAGTGCTGTGGCCTGGAGCAACAAATCTGAC*<br>*TTTGCATGTGCAAACGCCTTCAACAACAGCATTATTCCAGAAGACACCTTCTTCCCCAGCCCAGAAAGTTC*<br>*CTGTGATGTCAAGCTGGTCGAGAAAAGCTTTGAAACAGATACGAACCTAAACTTTCAAAACCTGTCAGTGA*<br>*TTGGGTTCCGAATCCTCCTCCTGAAAGTGGCCGGGTTTAATCTGCTCATGACGCTGCGGCTGTGGTCCAGC*<br>*tga*TCTAGA<br><br>• **TCR beta-chain variable region**<br>• *TCR beta-chain constant region*<br>• Linker<br>• 2A peptide<br>• **TCR alpha-chain variable region**<br>• TCR alpha-chain constant region	
TCR CD4-CMV#1	**GAATTC**AGCGGCCGCACC**ATGGGCTGCAGGCTCCTCTGCTGTGTGGTCTTCTGCCTCCTCCAAGCAGGTCCCTTGGACACAGCTG**<br>**TTTCCCAGACTCCAAAATACCTGGTCACACAGATGGGAAACGACAAGTCCATTAAATGTGAACAAAATCTGGGCCATGATACTAT**<br>**GTATTGGTATAAACAGGACTCTAAGAAATTTCTGAAGATAATGTTTAGCTACAATAATAAGGAGCTCATTATAAATGAAACAGTT**<br>**CCAAATCGCTTCTCACCTAAATCTCCAGACAAAGCTCACTTAAATCTTCACATCAATTCCCTGGAGCTTGGTGACTCTGCTGTGT**<br>**ATTTCTGTGCCAGCAGTCAAGAGAAACGGGGGGCTTTCTTCGGGCCAGGGACACGGCTCACCGTGGTTGAGGAC***CTGAACAAGGT*<br>*GTTCCCACCCGAGGTCGCTGTGTTTGAGCCATCAGAAGCAGAGATCTCCCACACCCAAAAGGCCACACTGGTGTGCCTGGCCACA*<br>*GGCTTCTTCCCCGACCACGTGGAGCTGAGCTGGTGGGTGAATGGGAAGGAGGTGCACAGTGGGGTCAGCACGGACCCGCAGCCCC*<br>*TCAAGGAGCAGCCCGCCCTCAATGACTCCAGATACTGCCTGAGCAGCCGCCTGAGGGTTTCGGCCACCTTCTGGCAGAACCCCCG*<br>*CAACCACTTCCGCTGTCAAGTCCAGTTCTACGGGCTCTCGGAGAATGACGAGTGGACCCAGGATAGGGCCAAACCCGTCACCCAG*<br>*ATCGTCAGCGCCGAGGCCTGGGGTAGAGCAGACTGTGGCTTTACCTCGGTGTCCTACCAGCAAGGGGTCCTGTCTGCCACCATCC*<br>*TCTATGAGATCCTGCTAGGGAAGGCCACCCTGTATGCTGTGCTGGTCAGCGCCCTTGTGTTGATGGCCATGGTCAAGAGAAAGGA*<br>*TTTC*AGGAGGAGGAGGAGCGGCAGTGGACTCGAGGTGAAACAGACTTTGAATTTTGACCTTCTCAAGTTGGCGGGAGACGTGGAG<br>TCCAACCCAGGGCCC**ATGGAGACACTCTTGGGCCTGCTTATCCTTTGGCTGCAGCTGCAATGGGTGAGCAGCAAACAGGAGGTGA**<br>**CGCAGATTCCTGCAGCTCTGAGTGTCCCAGAAGGAGAAAACTTGGTTCTCAACTGCAGTTTCACTGATAGCGCTATTTACAACCT**<br>**CCAGTGGTTTAGGCAGGACCCTGGGAAAGGACTCACATCTCTGTTGCTTATTCAGTCAAGTCAGAGAGAGCAAACAAGTGGAAGA**<br>**CTTAATGCCTCGCTGGATAAATCATCAGGACGTAGTACTTTATACATTGCAGCTTCTCAGCCTGGTGACTCAGCCACCTACCTCT**<br>**GTGCTGTTAAAGACAATGATATGCGTTTTGGAGCCGGAACCAGGCTCACTGTAAAACCTAATATCCAGAACCCTGACCCTGCC*GT***<br>***ATAC****CAGCTGAGAGACTCTAAATCCAGTGACAAGTCTGTCTGCCTATTCACCGATTTTGATTCTCAAACAAATGTGTCACAAAGT*<br>*AAGGATTCTGATGTGTATATCACAGACAAAACTGTGCTAGACATGAGGTCTATGGACTTCAAGAGCAACAGTGCTGTGGCCTGGA*<br>*GCAACAAATCTGACTTTGCATGTGCAAACGCCTTCAACAACAGCATTATTCCAGAAGACACCTTCTTCCCCAGCCCAGAAAGTTC*<br>*CTGTGATGTCAAGCTGGTCGAGAAAAGCTTTGAAACAGATACGAACCTAAACTTTCAAAACCTGTTAGTGATTGTGTTGCGAATC*<br>*CTCCTCCTGAAAGTGGTCGGGTTTAATCTGCTCATGACGCTGCGGCTGTGGTCCAGCTGATCTAGA*<br><br>• **TCR beta-chain variable region**<br>• *TCR beta-chain constant region*<br>• 2A peptide<br>• **TCR alpha-chain variable region**<br>• TCR alpha-chain constant region	**MGCRLLCCVVFCLLQAGPLDTAVSQTPKYLV**<br>**TQMGNDKSIKCEQNLGHDTMYWYKQDSKKFL**<br>**KIMFSYNNKELIINETVPNRFSPKSPDKAHL**<br>**NLHINSLELGDSAVYFCASSQEKRGAFFGPG**<br>**TRLTVVED**LNKVFPPEVAVFEPSEAEISHTQ<br>KATLVCLATGFFPDHVELSWWVNGKEVHSGV<br>STDPQPLKEQPALNDSRYCLSSRLRVSATFW<br>QNPRNHFRCQVQFYGLSENDEWTQDRAKPVT<br>QIVSAEAWGRADCGFTSVSYQQGVLSATILY<br>EILLGKATLYAVLVSALVLMAMVKRKDFRRR<br>RS**GSGLEVKQTLNFDLLKLAGDVESNPGPME**<br>**TLLGLLILWLQLQWVSSKQEVTQIPAALSVP**<br>**EGENLVLNCSFTDSAIYNLQWFRQDPGKGLT**<br>**SLLLIQSSQREQTSGRLNASLDKSSGRSTLY**<br>**IAASQPGDSATYLCAVKDNDMRFGAGTRLTV**<br>**KPNIQNPDPA***VYQLRDSKSSDKSVCLFTDFD*<br>*SQTNVSQSKDSDVYITDKTVLDMRSMDFKSN*<br>*SAVAWSNKSDFACANAFNNSIIPEDTFFPSP*<br>*ESSCDVKLVEKSFETDTNLNFQNLLVIVLRI*<br>*LLLKVVGFNLLMTLRLWSS*

TCR-NYESO-KFJ05	GAATTCAGCGGCCGCACC**ATGGGAATCAGGCTCCTGTGTCGTGTGGCCTTTTGTTTCCTGGCTGTAGGCCTCGTAGATGTGAAAG**<br>**TAACCCAGAGCTCGAGATATCTAGTCAAAAGGACGGGAGAGAAAGTTTTTCTGGAATGTGTCCAGGATATGGACCATGAAAATAT**<br>**GTTCTGGTATCGACAAGACCCAGGTCTGGGGCTACGGCTGATCTATTTCTCATATGATGTTAAAATGAAAGAAAAAGGAGATATT**<br>**CCTGAGGGGTACAGTGTCTCTAGAGAGAAGAAGGAGCGCTTCTCCCTGATTCTGGAGTCCGCCAGCACCAACCAGACATCTATGT**<br>**ACCTCTGCGCGAGCAGCCAGCGCCAGGAAGGCGATACCCAGTATTTTGGCCCAGGCACCCGGCTGACAGTGCTCGAGGAC**CTGAA<br>CAAGGTGTTCCCACCCGAGGTCGCTGTGTTTGAGCCATCAGAAGCAGAGATCTCCCACACCCAAAAGGCCACACTGGTGTGCCTG<br>GCCACAGGCTTCTTCCCCGACCACGTGGAGCTGAGCTGGTGGGTGAATGGGAAGGAGGTGCACAGTGGGGTCAGCACGGACCCGC<br>AGCCCCTCAAGGAGCAGCCCGCCCTCAATGACTCCAGATACTGCCTGAGCAGCCGCCTGAGGGTTTCGGCCACCTTCTGGCAGAA<br>CCCCCGCAACCACTTCCGCTGTCAAGTCCAGTTCTACGGGCTCTCGGAGAATGACGAGTGGACCCAGGATAGGGCCAAACCCGTC<br>ACCCAGATCGTCAGCGCCGAGGCCTGGGGTAGAGCAGACTGTGGCTTTACCTCGGTGTCCTACCAGCAAGGGGTCCTGTCTGCCA<br>CCATCCTCTATGAGATCCTGCTAGGGAAGGCCACCCTGTATGCTGTGCTGGTCAGCGCCCTTGTGTTGATGGCCATGGTCAAGAG<br>AAAGGATTTC**AGG**AGG**AGG**AGG**AGC**GGC**AGT**GGA**CTCGAGGTGAAACAGACTTTGAATTTTGACCTTCTCAAGTTGGCGGGAGAC**<br>**GTGGAGTCCAACCCAGGGCCC**ATGAGGCAAGTGGCGAGAGTGATCGTGTTCCTGACCCTGAGTACTTTGAGCCTTGCTAAGACCA<br>CCCAGCCCATCTCCATGGACTCATATGAAGGACAAGAAGTGAACATAACCTGTAGCCACAACAACATTGCTACAAATGATTATAT<br>CACGTGGTACCAACAGTTTCCCAGCCAAGGACCACGATTTATTATTCAAGGATACAAGACAAAAGTTACAAACGAAGTGGCCTCC<br>CTGTTTATCCCTGCCGACAGAAAGTCCAGCACTCTGAGCCTGCCCCGGGTTTCCCTGAGCGACACTGCTGTGTACTACTGCCTGG<br>TGGGCGAAATTCTGGATAACTTTAACAAATTTTATTTTGGATCTGGGACCAAACTCAATGTAAAACCAAATATCCAGAACCCTGA<br>CCCTGCC**GTATACCAGCTG**AGAGACTCTAAATCCAGTGACAAGTCTGTCTGCCTATTCACCGATTTTGATTCTCAAACAAATGTG<br>TCACAAAGTAAGGATTCTGATGTGTATATCACAGACAAAACTGTGCTAGACATGAGGTCTATGGACTTCAAGAGCAACAGTGCTG<br>TGGCCTGGAGCAACAAATCTGACTTTGCATGTGCAAACGCCTTCAACAACAGCATTATTCCAGAAGACACCTTCTTCCCCAGCCC<br>AGAAAGTTCCTGTGATGTCAAGCTGGTCGAGAAAAGCTTTGAAACAGATACGAACCTAAACTTTCAA**AAC**CTG**TTA**GTG**ATT**GTG<br>**TTG**CGA**ATC**CTC**CTC**CTG**AAA**GTG**GTC**GGG**TTT**AAT**CTG**CTC**ATG**ACG**CTG**CGG**CTG**TGG**TCC**AGC**TGA**<br><br>• **TCR beta-chain variable region**<br>• *TCR beta-chain constant region*<br>• 2A peptide<br>• **TCR alpha-chain variable region**<br>• TCR alpha-chain constant region	**MGIRLLCRVAFCFLAVGLVDVKVTQSSRYLV**<br>**KRTGEKVFLECVQDMDHENMFWYRQDPGLGL**<br>**RLIYFSYDVKMKEKGDIPEGYSVSREKKERF**<br>**SLILESASTNQTSMYLCASSQRQEGDTQYFG**<br>**PGTRLTVLED**LNKVFPPEVAVFEPSEAEISH<br>TQKATLVCLATGFFPDHVELSWWVNGKEVHS<br>GVSTDPQPLKEQPALNDSRYCLSSRLRVSAT<br>FWQNPRNHFRCQVQFYGLSENDEWTQDRAKP<br>VTQIVSAEAWGRADCGFTSVSYQQGVLSATI<br>LYEILLGKATLYAVLVSALVLMAMVKRKDFR<br>RRRSGSGLEVKQTLNFDLLKLAGDVESNPGP<br>MRQVARVIVFLTLSTLSLAKTTQPISMDSYE<br>GQEVNITCSHNNIATNDYITWYQQFPSQGPR<br>FIIQGYKTKVTNEVASLFIPADRKSSTLSLP<br>RVSLSDTAVYYCLVGEILDNFNKFYFGSGTK<br>LNVKPNIQNPDPAVYQLRDSKSSDKSVCLFT<br>DFDSQTNVSQSKDSDVYITDKTVLDMRSMDF<br>KSNSAVAWSNKSDFACANAFNNSIIPEDTFF<br>PSPESSCDVKLVEKSFETDTNLNFQNLLVIV<br>LRILLLKVVGFNLLMTLRLWSS
TCR-NYESO-KFJ15	GAATTCAGCGGCCGCACC**ATGAGCATCGGGCTCCTGTGCTGTGTGGCCTTTTCTCTCCTGTGGGCAAGTCCAGTGAATGCTGGTG**<br>**TCACTCAGACCCCAAAATTCCAGGTCCTGAAGACAGGACAGAGCATGACACTGCAGTGTGCCCAGGATATGAACCATAACTCCAT**<br>**GTACTGGTATCGACAAGACCCAGGCATGGGACTGAGGCTGATTTATTACTCAGCTTCTGAGGGTACCACTGACAAAGGAGAAGTC**<br>**CCCAATGGCTACAATGTCTCCAGATTAAACAAACGGGAGTTCTCGCTCAGGCTGGAGTCGGCTGCTCCCTCCCAGACATCTGTGT**<br>**ACTTCTGCGCGAGCCAGACCTTTAGCCCGGGCCAGACCCAGTTTGGGCCGGGCACCAGGCTCACGGTCACAGAGGA**CCTGAACAA<br>GGTGTTCCCACCCGAGGTCGCTGTGTTTGAGCCATCAGAAGCAGAGATCTCCCACACCCAAAAGGCCACACTGGTGTGCCTGGCC<br>ACAGGCTTCTTCCCCGACCACGTGGAGCTGAGCTGGTGGGTGAATGGGAAGGAGGTGCACAGTGGGGTCAGCACGGACCCGCAGC<br>CCCTCAAGGAGCAGCCCGCCCTCAATGACTCCAGATACTGCCTGAGCAGCCGCCTGAGGGTTTCGGCCACCTTCTGGCAGAACCC<br>CCGCAACCACTTCCGCTGTCAAGTCCAGTTCTACGGGCTCTCGGAGAATGACGAGTGGACCCAGGATAGGGCCAAACCCGTCACC<br>CAGATCGTCAGCGCCGAGGCCTGGGGTAGAGCAGACTGTGGCTTTACCTCGGTGTCCTACCAGCAAGGGGTCCTGTCTGCCACCA<br>TCCTCTATGAGATCCTGCTAGGGAAGGCCACCCTGTATGCTGTGCTGGTCAGCGCCCTTGTGTTGATGGCCATGGTCAAGAGAAA<br>GGATTTC**AGGAGGAGGAGGAGCGGCAGTGGACTCGAGGTGAAACAGACTTTGAATTTTGACCTTCTCAAGTTGGCGGGAGACGTG**<br>**GAGTCCAACCCAGGGCCC**ATGGAAACTCTCCTGGGAGTGTCTTTGGTGATTCTATGGCTTCAACTGGCTAGGGTGAACAGTCAAC<br>AGGGAGAAGAGGATCCTCAGGCCTTGAGCATCCAGGAGGGTGAAAATGCCACCATGAACTGCAGTTACAAAACTAGTATAAACAA<br>TTTACAGTGGTATAGACAAAATTCAGGTAGAGGCCTTGTCCACCTAATTTTAATACGTTCAAATGAAAGAGAGAAACACAGTGGA<br>AGATTAAGAGTCACGCTTGACACTTCCAAGAAAAGCAGTTCCTTGTTGATCACGGCTTCCCGGGCAGCAGACACTGCTTCTTACT<br>TCTGCGCGACCGGCAACGATTATAAACTGAGCTTTGGAGCCGGAACCACAGTAACTGTAAGAGCAAATATCCAGAACCCTGACCC<br>TGCC**GTATACCAGCTG**AGAGACTCTAAATCCAGTGACAAGTCTGTCTGCCTATTCACCGATTTTGATTCTCAAACAAATGTGTCA<br>CAAAGTAAGGATTCTGATGTGTATATCACAGACAAAACTGTGCTAGACATGAGGTCTATGGACTTCAAGAGCAACAGTGCTGTGG<br>CCTGGAGCAACAAATCTGACTTTGCATGTGCAAACGCCTTCAACAACAGCATTATTCCAGAAGACACCTTCTTCCCCAGCCCAGA<br>AAGTTCCTGTGATGTCAAGCTGGTCGAGAAAAGCTTTGAAACAGATACGAACCTAAACTTTCAAAACCTGTTAGTGATTGTGTTG<br>CGAATCCTCCTCCTGAAAGTGGTCGGGTTTAATCTGCTCATGACGCTGCGGCTGTGGTCCAGCTGA<br><br>• **TCR beta-chain variable region**<br>• *TCR beta-chain constant region*	**MSIGLLCCVAFSLLWASPVNAGVTQTPKFQV**<br>**LKTGQSMTLQCAQDMNHNSMYWYRQDPGMGL**<br>**RLIYYSASEGTTDKGEVPNGYNVSRLNKREF**<br>**SLRLESAAPSQTSVYFCASQTFSPGQTQFGP**<br>**GTRLTVTED**LNKVFPPEVAVFEPSEAEISHT<br>QKATLVCLATGFFPDHVELSWWVNGKEVHSG<br>VSTDPQPLKEQPALNDSRYCLSSRLRVSATF<br>WQNPRNHFRCQVQFYGLSENDEWTQDRAKPV<br>TQIVSAEAWGRADCGFTSVSYQQGVLSATIL<br>YEILLGKATLYAVLVSALVLMAMVKRKDFRR<br>RRSGSGLEVKQTLNFDLLKLAGDVESNPGPM<br>ETLLGVSLVILWLQLARVNSQQGEEDPQALS<br>IQEGENATMNCSYKTSINNLQWYRQNSGRGL<br>VHLILIRSNEREKHSGRLRVTLDTSKKSSSL<br>LITASRAADTASYFCATGNDYKLSFGAGTTV<br>TVRANIQNPDPAVYQLRDSKSSDKSVCLFTD<br>FDSQTNVSQSKDSDVYITDKTVLDMRSMDFK<br>SNSAVAWSNKSDFACANAFNNSIIPEDTFFP<br>SPESSCDVKLVEKSFETDTNLNFQNLLVIVL<br>RILLLKVVGFNLLMTLRLWSS

	• 2A peptide<br>• **TCR alpha-chain variable region**<br>• TCR alpha-chain constant region	
TCR-NYESO-KFJ37	GAATTCAGCGGCCGCACCATGGGCTTCAGGCTCCTCTGCTGTGTGGCCTTTTGTCTCCTGGGAGCAGGCCCAGTGGATTCTGGAG<br>TCACACAAACCCCAAAGCACCTGATCACAGCAACTGGACAGCGAGTGACGCTGAGATGCTCCCCTAGGTCTGGAGACCTCTCTGT<br>GTACTGGTACCAACAGAGCCTGGACCAGGGCCTCCAGTTCCTCATTCAGTATTATAATGGAGAAGAGAGAGCAAAAGGAAACATT<br>CTTGAACGATTCTCCGCACAACAGTTCCCTGACTTGCACTCTGAACTAAACCTGAGCTCTCTGGAGCTGGGGGACTCAGCTTTGT<br>ATTTCTGCGCGAGCAGCGGCGGCCATACCGGCAGCAACGAACAGTTTTTTGGGCCAGGGACACGGCTCACCGTGCTAGAGGACCT<br>GAACAAGGTGTTCCCACCCGAGGTCGCTGTGTTTGAGCCATCAGAAGCAGAGATCTCCCACACCCAAAAGGCCACACTGGTGTGC<br>CTGGCCACAGGCTTCTTCCCCGACCACGTGGAGCTGAGCTGGTGGGTGAATGGGAAGGAGGTGCACAGTGGGGTCAGCACGGACC<br>CGCAGCCCCTCAAGGAGCAGCCCGCCCTCAATGACTCCAGATACTGCCTGAGCAGCCGCCTGAGGGTTTCGGCCACCTTCTGGCA<br>GAACCCCCGCAACCACTTCCGCTGTCAAGTCCAGTTCTACGGGCTCTCGGAGAATGACGAGTGGACCCAGGATAGGGCCAAACCC<br>GTCACCCAGATCGTCAGCGCCGAGGCCTGGGGTAGAGCAGACTGTGGCTTTACCTCGGTGTCCTACCAGCAAGGGGTCCTGTCTG<br>CCACCATCCTCTATGAGATCCTGCTAGGGAAGGCCACCCTGTATGCTGTGCTGGTCAGCGCCCTTGTGTTGATGGCCATGGTCAA<br>GAGAAAGGATTTCAGGAGGAGGAGGAGCGGCAGTGGACTCGAGGTGAAACAGACTTTGAATTTTGACCTTCTCAAGTTGGCGGGA<br>GACGTGGAGTCCAACCCAGGGCCCATGAGGCAAGTGGCGAGAGTGATCGTGTTCCTGACCCTGAGTACTTTGAGCCTTGCTAAGA<br>CCACCCAGCCCATCTCCATGGACTCATATGAAGGACAAGAAGTGAACATAACCTGTAGCCACAACAACATTGCTACAAATGATTA<br>TATCACGTGGTACCAACAGTTTCCCAGCCAAGGACCACGATTTATTATTCAAGGATACAAGACAAAAGTTACAAACGAAGTGGCC<br>TCCCTGTTTATCCCTGCCGACAGAAAGTCCAGCACTCTGAGCCTGCCCCGGGTTTCCCTGAGCGACACTGCTGTGTACTACTGCC<br>TGGTGGTGGATCAGAAACTGGTGTTTGGAACTGGCACCCGACTTCTGGTCAGTCCAAATATCCAGAACCCTGACCCTGCCGTATA<br>CCAGCTGAGAGACTCTAAATCCAGTGACAAGTCTGTCTGCCTATTCACCGATTTTGATTCTCAAACAAATGTGTCACAAAGTAAG<br>GATTCTGATGTGTATATCACAGACAAAACTGTGCTAGACATGAGGTCTATGGACTTCAAGAGCAACAGTGCTGTGGCCTGGAGCA<br>ACAAATCTGACTTTGCATGTGCAAACGCCTTCAACAACAGCATTATTCCAGAAGACACCTTCTTCCCCAGCCCAGAAAGTTCCTG<br>TGATGTCAAGCTGGTCGAGAAAAGCTTTGAAACAGATACGAACCTAAACTTTCAAAACCTGTTAGTGATTGTGTTGCGAATCCTC<br>CTCCTGAAAGTGGTCGGGTTTAATCTGCTCATGACGCTGCGGCTGTGGTCCAGCTGA<br>• **TCR beta-chain variable region**<br>• *TCR beta-chain constant region*<br>• 2A peptide<br>• **TCR alpha-chain variable region**<br>• TCR alpha-chain constant region	**MGFRLLCCVAFCLLGAGPVDSGVTQTPKHLI**<br>**TATGQRVTLRCSPRSGDLSVYWYQQSLDQGL**<br>**QFLIQYYNGEERAKGNILERFSAQQFPDLHS**<br>**ELNLSSLELGDSALYFCASSGGHTGSNEQFF**<br>**GPGTRLTVLED**LNKVFPPEVAVFEPSEAEIS<br>HTQKATLVCLATGFFPDHVELSWWVNGKEVH<br>SGVSTDPQPLKEQPALNDSRYCLSSRLRVSA<br>TFWQNPRNHFRCQVQFYGLSENDEWTQDRAK<br>PVTQIVSAEAWGRADCGFTSVSYQQGVLSAT<br>ILYEILLGKATLYAVLVSALVLMAMVKRKDF<br>RRRRSGSGLEVKQTLNFDLLKLAGDVESNPG<br>PMRQVARVIVFLTLSTLSLAKTTQPISMDSY<br>EGQEVNITCSHNNIATNDYITWYQQFPSQGP<br>RFIIQGYKTKVTNEVASLFIPADRKSSTLSL<br>PRVSLSDTAVYYCLVVDQKLVFGTGTRLLVS<br>PNIQNPDPAVYQLRDSKSSDKSVCLFTDFDS<br>QTNVSQSKDSDVYITDKTVLDMRSMDFKSNS<br>AVAWSNKSDFACANAFNNSIIPEDTFFPSPE<br>SSCDVKLVEKSFETDTNLNFQNLLVIVLRIL<br>LLKVVGFNLLMTLRLWSS
TCR-LMP2A-C1	GAATTCAGCGGCCGCACCATGAGCCTCGGGCTCCTGTGCTGTGGGGTCTTTTCTCTCCTGTGGGCAGGTCCAGTGAATGCTGGTG<br>TCACTCAGACCCCAAAATTCCGGGTCCTGAAGACAGGACAGAGCATGACACTGCTGTGTGCCCAGGATATGAACCATGAATACAT<br>GTACTGGTATCGACAAGACCCAGGCATGGGGCTGAGGCTGATTCATTACTCAGTTGGTGAGGGTACAACTGCCAAAGGAGAGGTC<br>CCTGATGGCTACAATGTCTCCAGATTAAAAAAACAGAATTTCCTGCTGGGGTTGGAGTCGGCTGCTCCCTCCCAAACATCTGTGT<br>ACTTCTGCGCGAGCAGCCCGGAAGGCGTGTTTAACGAACAGTTTTTCGGGCCAGGGACACGGCTCACCGTGCTAGAGGACCTGAA<br>CAAGGTGTTCCCACCCGAGGTCGCTGTGTTTGAGCCATCAGAAGCAGAGATCTCCCACACCCAAAAGGCCACACTGGTGTGCCTG<br>GCCACAGGCTTCTTCCCCGACCACGTGGAGCTGAGCTGGTGGGTGAATGGGAAGGAGGTGCACAGTGGGGTCAGCACGGACCCGC<br>AGCCCCTCAAGGAGCAGCCCGCCCTCAATGACTCCAGATACTGCCTGAGCAGCCGCCTGAGGGTTTCGGCCACCTTCTGGCAGAA<br>CCCCCGCAACCACTTCCGCTGTCAAGTCCAGTTCTACGGGCTCTCGGAGAATGACGAGTGGACCCAGGATAGGGCCAAACCCGTC<br>ACCCAGATCGTCAGCGCCGAGGCCTGGGGTAGAGCAGACTGTGGCTTTACCTCGGTGTCCTACCAGCAAGGGGTCCTGTCTGCCA<br>CCATCCTCTATGAGATCCTGCTAGGGAAGGCCACCCTGTATGCTGTGCTGGTCAGCGCCCTTGTGTTGATGGCCATGGTCAAGAG<br>AAAGGATTTCAGGAGGAGGAGGAGCGGCAGTGGACTCGAGGTGAAACAGACTTTGAATTTTGACCTTCTCAAGTTGGCGGGAGAC<br>GTGGAGTCCAACCCAGGGCCCATGGAGACTCTCCTGAAAGTGCTTTCAGGCACCTTGTTGTGGCAGTTGACCTGGGTGAGAAGCC<br>AACAACCAGTGCAGAGTCCTCAAGCCGTGATCCTCCGAGAAGGGGAAGATGCTGTCATCAACTGCAGTTCCTCCAAGGCTTTATA<br>TTCTGTACACTGGTACAGGCAGAAGCATGGTGAAGCACCCGTCTTCCTGATGATATTACTGAAGGGTGGAGAACAGAAGGGTCAT<br>GAAAAAATATCTGCTTCATTTAATGAAAAAAAGCAGCAAAGCTCCCTGTACCTTACGGCCTCCCAGCTCAGTTACTCAGGAACCT<br>ACTTCTGCGGCACCGGCAGCGGCGGCGGCGCGGATGGCCTGACCTTTGGCAAAGGGACTCATCTAATCATCCAGCCCTATATCCA<br>GAACCCTGACCCTGCCGTATACCAGCTGAGAGACTCTAAATCCAGTGACAAGTCTGTCTGCCTATTCACCGATTTTGATTCTCAA<br>ACAAATGTGTCACAAAGTAAGGATTCTGATGTGTATATCACAGACAAAACTGTGCTAGACATGAGGTCTATGGACTTCAAGAGCA<br>ACAGTGCTGTGGCCTGGAGCAACAAATCTGACTTTGCATGTGCAAACGCCTTCAACAACAGCATTATTCCAGAAGACACCTTCTT<br>CCCCAGCCCAGAAAGTTCCTGTGATGTCAAGCTGGTCGAGAAAAGCTTTGAAACAGATACGAACCTAAACTTTCAAAACCTGTTA	**MSLGLLCCGVFSLLWAGPVNAGVTQTPKFRV**<br>**LKTGQSMTLLCAQDMNHEYMYWYRQDPGMGL**<br>**RLIHYSVGEGTTAKGEVPDGYNVSRLKKQNF**<br>**LLGLESAAPSQTSVYFCASSPEGVFNEQFFG**<br>**PGTRLTVLED**LNKVFPPEVAVFEPSEAEISH<br>TQKATLVCLATGFFPDHVELSWWVNGKEVHS<br>GVSTDPQPLKEQPALNDSRYCLSSRLRVSAT<br>FWQNPRNHFRCQVQFYGLSENDEWTQDRAKP<br>VTQIVSAEAWGRADCGFTSVSYQQGVLSATI<br>LYEILLGKATLYAVLVSALVLMAMVKRKDFR<br>RRRSGSGLEVKQTLNFDLLKLAGDVESNPGP<br>METLLKVLSGTLLWQLTWVRSQQPVQSPQAV<br>ILREGEDAVINCSSSKALYSVHWYRQKHGEA<br>PVFLMILLKGGEQKGHEKISASFNEKKQQSS<br>LYLTASQLSYSGTYFCGTGSGGGADGLTFGK<br>GTHLIIQPYIQNPDPAVYQLRDSKSSDKSVC<br>LFTDFDSQTNVSQSKDSDVYITDKTVLDMRS<br>MDFKSNSAVAWSNKSDFACANAFNNSIIPED

	GTGATTGTGTTGCGAATCCTCCTCCTGAAAGTGGTCGGGTTTAATCTGCTCATGACGCTGCGGCTGTGGTCCAGCTGA<br>• **TCR beta-chain variable region**<br>• *TCR beta-chain constant region*<br>• 2A peptide<br>• **TCR alpha-chain variable region**<br>• TCR alpha-chain constant region	TFFPSPESSCDVKLVEKSFETDTNLNFQNLL<br>VIVLRILLLKVVGFNLLMTLRLWSS
TCR-LMP2A-C2	GAATTCAGCGGCCGCACCATGAGCCTCGGGCTCCTGTGCTGTGGGGTCTTTTCTCTCCTGTGGGCAGGTCCAGTGAATGCTGGT<br>GTCACTCAGACCCCAAAATTCCGGGTCCTGAAGACAGGACAGAGCATGACACTGCTGTGTGCCCAGGATATGAACCATGAATACA<br>TGTACTGGTATCGACAAGACCCAGGCATGGGGCTGAGGCTGATTCATTACTCAGTTGGTGAGGGTACAACTGCCAAAGGAGAGGT<br>CCCTGATGGCTACAATGTCTCCAGATTAAAAAAACAGAATTTCCTGCTGGGGTTGGAGTCGGCTGCTCCCTCCCAAACATCTGTG<br>TACTTCTGCGCGAGCAGCTATGGCATTTATGAACAGTTTTTCGGGCCAGGGACACGGCTCACCGTGCTAGAGGACCTGAACAAGG<br>TGTTCCCACCCGAGGTCGCTGTGTTTGAGCCATCAGAAGCAGAGATCTCCCACACCCAAAAGGCCACACTGGTGTGCCTGGCCAC<br>AGGCTTCTTCCCCGACCACGTGGAGCTGAGCTGGTGGGTGAATGGGAAGGAGGTGCACAGTGGGGTCAGCACGGACCCGCAGCCC<br>CTCAAGGAGCAGCCCGCCCTCAATGACTCCAGATACTGCCTGAGCAGCCGCCTGAGGGTTTCGGCCACCTTCTGGCAGAACCCCC<br>GCAACCACTTCCGCTGTCAAGTCCAGTTCTACGGGCTCTCGGAGAATGACGAGTGGACCCAGGATAGGGCCAAACCCGTCACCCA<br>GATCGTCAGCGCCGAGGCCTGGGGTAGAGCAGACTGTGGCTTTACCTCGGTGTCCTACCAGCAAGGGGTCCTGTCTGCCACCATC<br>CTCTATGAGATCCTGCTAGGGAAGGCCACCCTGTATGCTGTGCTGGTCAGCGCCCTTGTGTTGATGGCCATGGTCAAGAGAAAGG<br>ATTTCAGGAGGAGGAGGAGCGGCAGTGGACTCGAGGTGAAACAGACTTTGAATTTTGACCTTCTCAAGTTGGCGGGAGACGTGGA<br>GTCCAACCCAGGGCCCATGGAGACTCTCCTGAAAGTGCTTTCAGGCACCTTGTTGTGGCAGTTGACCTGGGTGAGAAGCCAACAA<br>CCAGTGCAGAGTCCTCAAGCCGTGATCCTCCGAGAAGGGGAAGATGCTGTCATCAACTGCAGTTCCTCCAAGGCTTTATATTCTG<br>TACACTGGTACAGGCAGAAGCATGGTGAAGCACCCGTCTTCCTGATGATATTACTGAAGGGTGGAGAACAGAAGGGTCATGAAAA<br>AATATCTGCTTCATTTAATGAAAAAAAGCAGCAAAGCTCCCTGTACCTTACGGCCTCCCAGCTCAGTTACTCAGGAACCTACTTC<br>TGCGGCACCGAAGATGGCCGCGGCGGCGCGGATGGCCTGACCTTTGGCAAAGGGACTCATCTAATCATCCAGCCCTATATCCAGA<br>ACCCTGACCCTGCCGTATACCAGCTGAGAGACTCTAAATCCAGTGACAAGTCTGTCTGCCTATTCACCGATTTTGATTCTCAAAC<br>AAATGTGTCACAAAGTAAGGATTCTGATGTGTATATCACAGACAAAACTGTGCTAGACATGAGGTCTATGGACTTCAAGAGCAAC<br>AGTGCTGTGGCCTGGAGCAACAAATCTGACTTTGCATGTGCAAACGCCTTCAACAACAGCATTATTCCAGAAGACACCTTCTTCC<br>CCAGCCCAGAAAGTTCCTGTGATGTCAAGCTGGTCGAGAAAAGCTTTGAAACAGATACGAACCTAAACTTTCAAAACCTGTTAGT<br>GATTGTGTTGCGAATCCTCCTCCTGAAAGTGGTCGGGTTTAATCTGCTCATGACGCTGCGGCTGTGGTCCAGCTGA<br>• **TCR beta-chain variable region**<br>• *TCR beta-chain constant region*<br>• 2A peptide<br>• **TCR alpha-chain variable region**<br>• TCR alpha-chain constant region	MSLGLLCCGVFSLLWAGPVNAGVTQTPKFRV<br>LKTGQSMTLLCAQDMNHEYMYWYRQDPGMGL<br>RLIHYSVGEGTTAKGEVPDGYNVSRLKKQNF<br>LLGLESAAPSQTSVYFCASSYGIYEQFFGPG<br>TRLTVLEDLNKVFPPEVAVFEPSEAEISHTQ<br>KATLVCLATGFFPDHVELSWWVNGKEVHSGV<br>STDPQPLKEQPALNDSRYCLSSRLRVSATFW<br>QNPRNHFRCQVQFYGLSENDEWTQDRAKPVT<br>QIVSAEAWGRADCGFTSVSYQQGVLSATILY<br>EILLGKATLYAVLVSALVLMAMVKRKDFRRR<br>RSGSGLEVKQTLNFDLLKLAGDVESNPGPME<br>TLLKVLSGTLLWQLTWVRSQQPVQSPQAVIL<br>REGEDAVINCSSSKALYSVHWYRQKHGEAPV<br>FLMILLKGGEQKGHEKISASFNEKKQQSSLY<br>LTASQLSYSGTYFCGTEDGRGGADGLTFGKG<br>THLIIQPYIQNPDPAVYQLRDSKSSDKSVCL<br>FTDFDSQTNVSQSKDSDVYITDKTVLDMRSM<br>DFKSNSAVAWSNKSDFACANAFNNSIIPEDT<br>FFPSPESSCDVKLVEKSFETDTNLNFQNLLV<br>IVLRILLLKVVGFNLLMTLRLWSS

Supplemental Figures

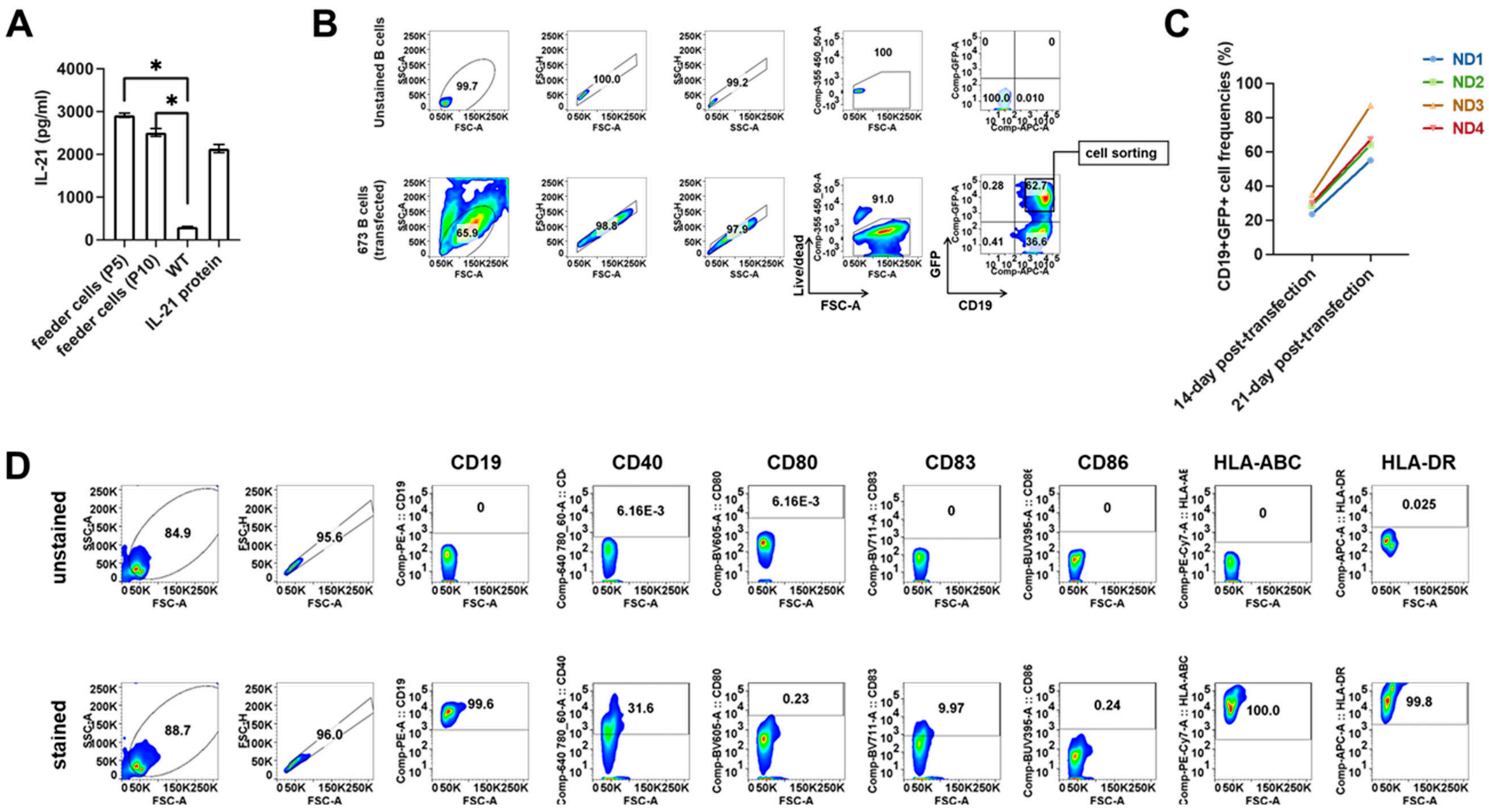

Supplementary Figure S1. Characterization of B cell immortalization. **(A**) IL-21 secretion from feeder cells. Supernatant samples from the genetically engineered feeder cells at 5th and 10th passage were subject to ELISA detecting the IL-21 secretion, with that from WT feeder cells serving as negative control. The IL-21 protein standards in the assay kit served as the positive control. (**B**) Flow cytometric gating strategy of GFP+CD19+ cells from the immortalized B cells. After retroviral transfection of BCL-6/BCL-XL and GFP-reporter genes, the transfected B cells were subjected to flow cytometric analysis for detecting the GFP+CD19+ cells, via using live/dead cell dye and anti-CD19 and examining GFP expression. Unstained ND B cells were used as the negative control for setting the gates of live cells and GFP+CD19+ cells. At day 14 post-transfection, the GFP+CD19+ cells were collected (with the proportion of the cells as indicated) using the BD FACSAria Fusion Cell Sorter. (**C**) Cell immortalization of B cells from different NDs. B cells from 4 NDs were immortalized, and the frequencies of GFP+CD19+ cells were detected via flow cytometric analyses at 14 and 21 days post retroviral transfection. (**D**) Flow cytometric gating strategy of immortalized B cell biomarker identification. B cell biomarkers were analyzed in ND B cells, along with immortalized B cells from ND#673 and Pt.1 as shown in Fig 1C. With unstained cells serving as the negative control, the analyzed cells were stained with antibodies against CD19, HLA-ABC, HLA-DR, CD40, CD80, CD63 and CD86. The expression of HLA-ABC, HLA-DR, CD40, CD80, CD63 and CD86 were gated from CD19+ cells. Related to Figure 1.

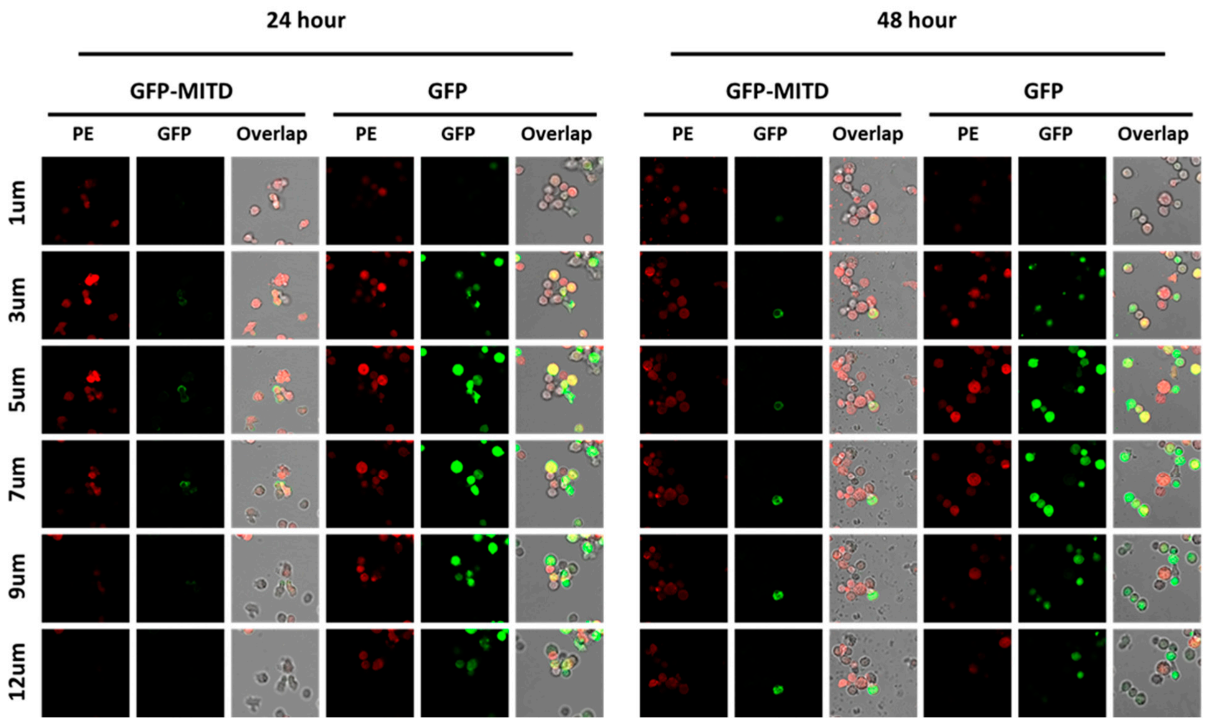

Supplementary Figure S2. Confocal imaging of immortalized B cells processing GFP-MITD-WT mRNA. mRNA of GFP or GFP-MITD-WT was electroporated into immortalized B cells, and confocal imaging was conducted at 24- and 48-hours post-mRNA transduction. A z-series imaging was conducted and visualization of the cells in different layers were provided respectively at 24 and 48 hours post mRNA electroporation. Related to Figure 2.

A

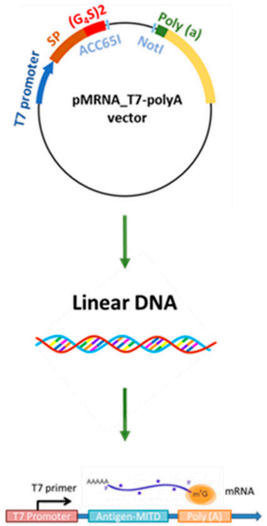

B

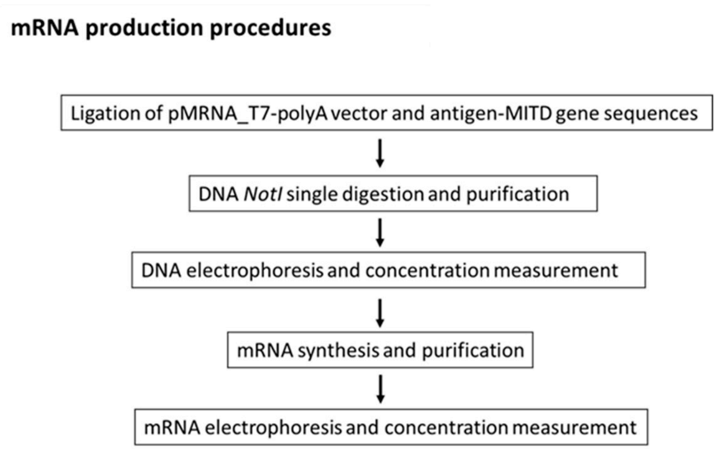

C

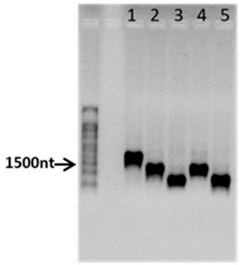

No	Gene-MITD WT	mRNA Conentration (ng/ul)	260/280	260/230
1	CMV-MITD-WT	2395.9	2.09	2.16
2	NEF-MITD-WT	2474.2	2.23	2.16
3	S2-MITD-WT	2953	2.28	2.15
4	S10-MITD-WT	3300.2	2.22	2.15
5	L2-MITD-WT	2886	2.25	2.41

D

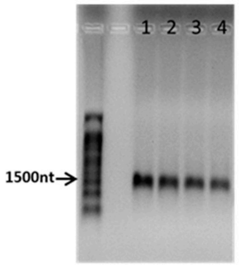

No.	CMV-MITD variants	mRNA Conentration (ng/ul)	260/280	260/230
1	CMV-MITD-Y320E	1953.6	2.12	2.16
2	CMV-MITD-S335E	2078.7	2.12	2.16
3	CMV-MITD-Y320E/S335E	2105.5	2.27	2.15
4	CMV-MITD-WT	2027.7	2.26	2.15

Supplementary Figure S3. Production of mRNA of CMV gene conjugated with MITD. (A-B) General schema of mRNA production. pMRNA_T7-polyA vector was used for ligation of the CMV gene and MITD sequences. (**C)** mRNA synthesis was conducted from five vector templates, containing MITD-WT and respectively CMVpp65-full length gene, Nef full length gene, S2 sequence, L2 sequence and S10 sequence. (**D)** mRNA synthesis was conducted from CMV-MITD vector templates, containing CMVpp65-full length gene and respectively MITD-WT, MITD-S335E, MITD-Y320E and MITD-Y320E/S335E gene. The ligated DNA was linearized via restriction enzyme Not**I** digestion, followed with DNA purification and confirmation with DNA gel electrophoresis. mRNA synthesis was conducted using HiScribe™ T7 ARCA mRNA Kit, with the concentration and purify confirmed by RNA denaturing gel electrophoresis and nanodrop. Related to Figure 2.

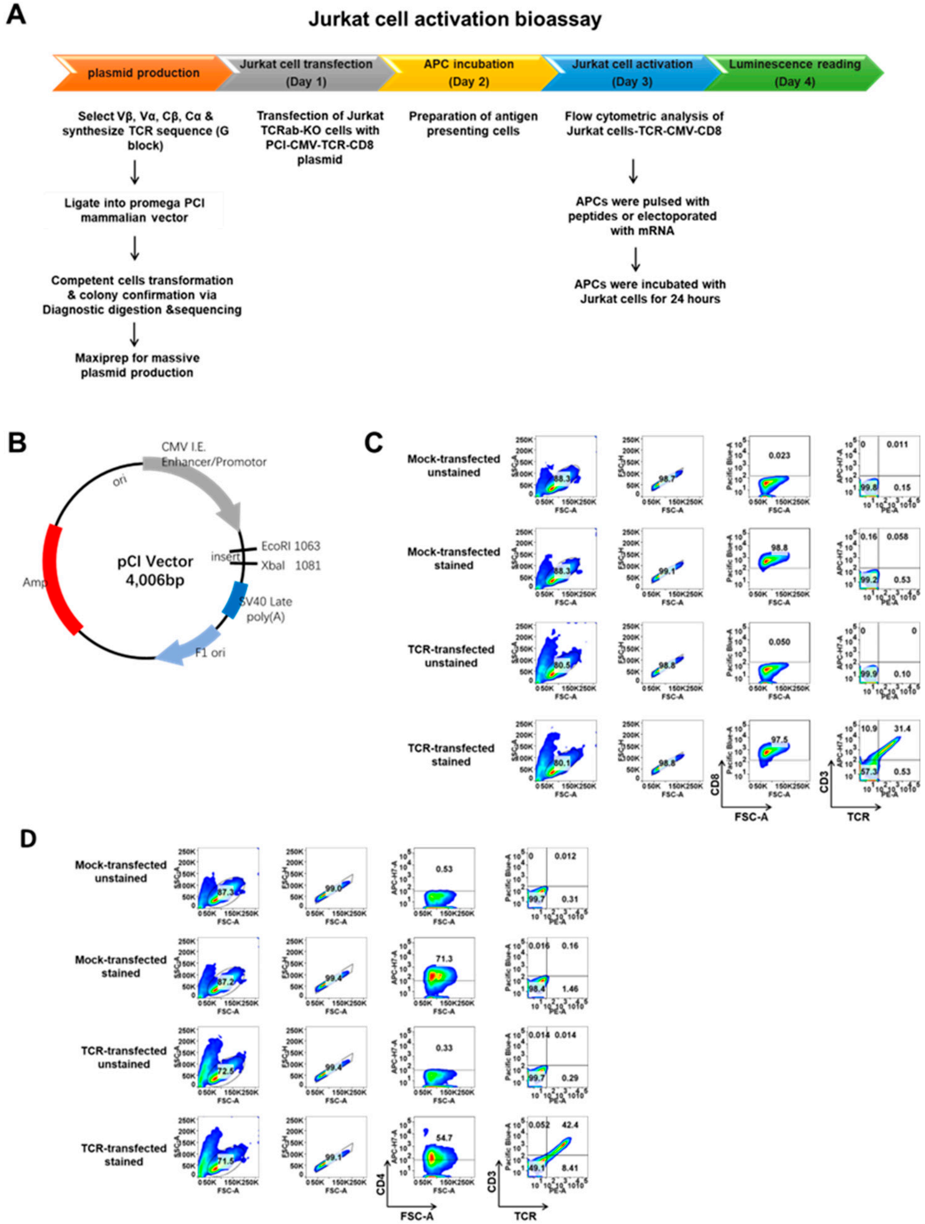
A
Jurkat cell activation bioassay
plasmid production
Jurkat cell transfection (Day 1)
APC incubation (Day 2)
Jurkat cell activation (Day 3)
Luminescence reading (Day 4)
Select Vβ, Vα, Cβ, Cα & synthesize TCR sequence (G block)
Ligate into promega PCI mammalian vector
Competent cells transformation & colony confirmation via Diagnostic digestion &sequencing
Maxiprep for massive plasmid production
Transfection of Jurkat TCRab-KO cells with PCI-CMV-TCR-CD8 plasmid
Preparation of antigen presenting cells
Flow cytometric analysis of Jurkat cells-TCR-CMV-CD8
APCs were pulsed with peptides or electoporated with mRNA
APCs were incubated with Jurkat cells for 24 hours
B
CMV I.E. Enhancer/Promotor
ori
insert
EcoRI 1063
XbaI 1081
pCI Vector 4,006bp
Amp
SV40 Late poly(A)
F1 ori
C
Mock-transfected unstained
Mock-transfected stained
TCR-transfected unstained
TCR-transfected stained
CD8
FSC-A
CD3
TCR
D
Mock-transfected unstained
Mock-transfected stained
TCR-transfected unstained
TCR-transfected stained
CD4
FSC-A
CD3
TCR

Supplementary Figure S4. Jurkat cell activation bioassay. (A) General schema of Jurkat cell activation bioassay. CMV pp65-specific TCR sequence was designed, synthesized, and ligated to the PCI mammalian vector (Promega). TCR deficient Jurkat cells were transfected with the engineered plasmid and subsequently stimulated with the B cells processing mRNA of antigens conjugated with MITD. (**B)** Template plasmid containing TCR sequences. PCI vector was digested with EcoRI and XbaI double digestion in the multiple cloning sites, followed with DNA purification. CMV pp65-specific TCR (TCR_{CD8}-CMV#4 and TCR_{CD4}-CMV#1) sequences were ligated into the PCI vector via the in-fusion cloning kit, followed with competent cell transformation and plating. Diagnostic sequencing was subsequently performed in picked colonies. (**C)** Flow cytometric analysis of Jurkat cells expressing CMV pp65-specific TCR. TCR-deficient Jurkat cells were transfected with engineered plasmid containing CMV pp65-specific TCR (TCR_{CD8}-CMV#4), and flow cytometric analysis was performed after 2 days confirming the expression of CD8, CD3 and TCR. (**D)** Flow cytometric analysis of Jurkat cells expressing CMV pp65-specific TCR. TCR-deficient Jurkat cells were transfected with engineered plasmid containing CMV pp65-specific TCR (TCR_{CD4}-CMV#1), and flow cytometric analysis was performed after 2 days confirming the expression of CD4, CD3 and TCR. Related to Figure 3.

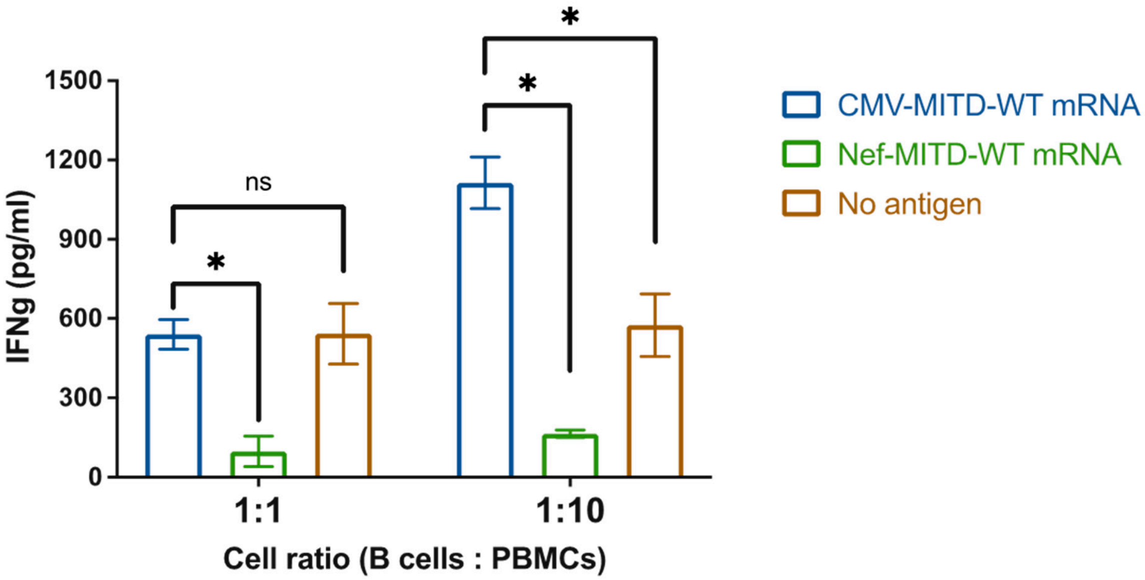

Supplementary Figure S5. **Optimization of B cell: PBMC quantity ratios for PBMC stimulation**. Autologous PBMCs were stimulated with immortalized ND #739 B cells respectively at 1:1 and 1:10 ratios (for two times). T cells were subsequently isolated from the stimulated PBMCs and co-incubated with immortalized B cells processing CMV-MITD-WT or Nef-MITD-WT mRNAs or with no antigens for 24 hours before IFNg release were detected. Related to Figure 6.

A

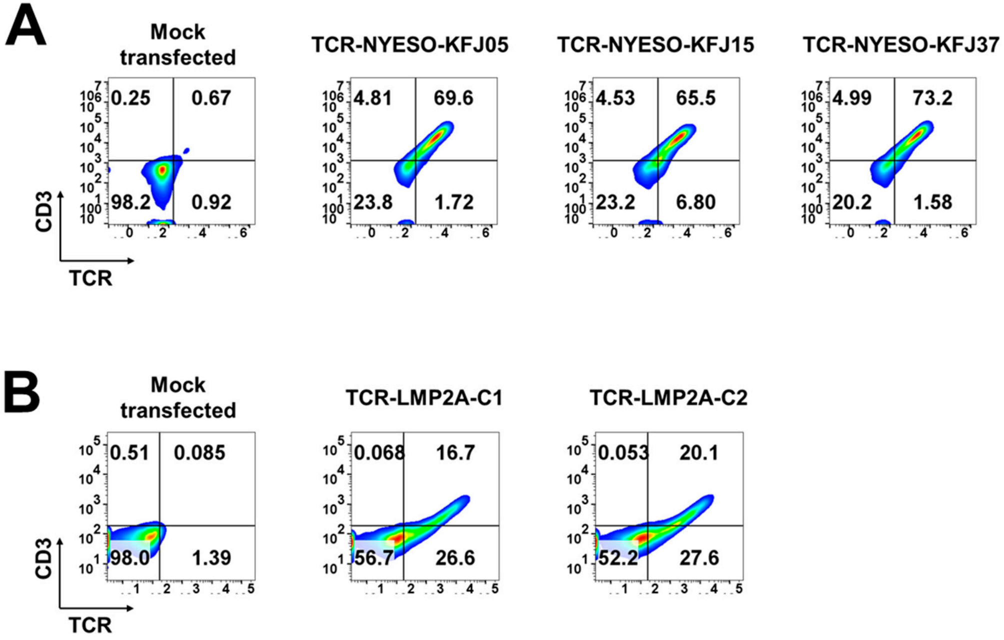

C

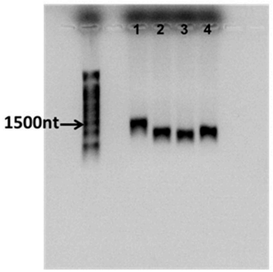

No.		mRNA concentration (ng/ul)	260/280	260/230
1	CMV-MITD-WT	1984.7	2.12	2.14
2	NY-ESO-1-MITD-WT	2107.8	2.23	2.35
3	LMP2A-MITD-WT	1162.0	2.21	2.39
4	NEF-MITD-WT	2164.3	2.28	2.44

Supplementary Figure S6. TCR expression on Jurkat cells and mRNA production. (A) Flow cytometric analysis of Jurkat cells expressing TCRs recognizing NY-ESO-1$_{60-72}$. TCRαβ-KO Jurkat cells were transfected with engineered plasmids respectively expressing TCR-NYESO-KFJ05, TCR-NYESO-KFJ15, and TCR-NYESO-KFJ37 and flow cytometric analysis was performed after 2 days confirming the expression of CD3 and TCR. (**B)** Flow cytometric analysis of Jurkat cells expressing TCRs recognizing LMP2A$_{484-493}$ epitope. TCRαβ-

KO Jurkat cells were transfected with engineered plasmids expressing TCR-LMP2A-C1 and TCR-LMP2A-C2 and flow cytometric analysis was performed after 2 days confirming the expression of CD3 and TCR. **(C)** mRNA synthesis was conducted from vector templates, respectively containing CMV-MITD-WT, NY-ESO-1-MITD-WT, LMP2A-MITD-WT, and Nef-MITD-WT. The ligated DNA was linearized via restriction enzyme NotI digestion, followed with DNA purification and confirmation with DNA gel electrophoresis. mRNA synthesis was conducted using HiScribe™ T7 ARCA mRNA Kit, with the concentration and purity confirmed by RNA denaturing gel electrophoresis and nanodrop. Related to Figure 7.
